# Intermolecular Electronic Coupling of Organic Units for Efficient Persistent Room‐Temperature Phosphorescence

**DOI:** 10.1002/anie.201509224

**Published:** 2016-01-06

**Authors:** Zhiyong Yang, Zhu Mao, Xuepeng Zhang, Depei Ou, Yingxiao Mu, Yi Zhang, Cunyuan Zhao, Siwei Liu, Zhenguo Chi, Jiarui Xu, Yuan‐Chun Wu, Po‐Yen Lu, Alan Lien, Martin R. Bryce

**Affiliations:** ^1^ PCFM Lab, GDHPPC Lab, Guangdong Engineering Technology Research Center for High-performance Organic and Polymer Photoelectric Functional FilmsState Key Laboratory of OEMTSchool of Chemistry and Chemical EngineeringSun Yat-sen UniversityGuangzhou510275China; ^2^ School of Chemistry and Chemical EngineeringSun Yat-sen UniversityGuangzhou510275China; ^3^ Shenzhen China Star Optoelectronics Technology Co., LtdChina; ^4^ Department of ChemistryDurham UniversityDurhamDH1 3LEUK

**Keywords:** intersystem crossing, organic materials, phosphorescence, photochemistry, spin–orbit coupling

## Abstract

Although persistent room‐temperature phosphorescence (RTP) emission has been observed for a few pure crystalline organic molecules, there is no consistent mechanism and no universal design strategy for organic persistent RTP (pRTP) materials. A new mechanism for pRTP is presented, based on combining the advantages of different excited‐state configurations in coupled intermolecular units, which may be applicable to a wide range of organic molecules. By following this mechanism, we have developed a successful design strategy to obtain bright pRTP by utilizing a heavy halogen atom to further increase the intersystem crossing rate of the coupled units. RTP with a remarkably long lifetime of 0.28 s and a very high quantum efficiency of 5 % was thus obtained under ambient conditions. This strategy represents an important step in the understanding of organic pRTP emission.

Room‐temperature phosphorescence (RTP) in organic materials is important in optoelectronic technologies, such as electroluminescence, molecular sensing, and time‐resolved bioimaging.^1^ However, unlike inorganic materials, which can possess afterglow or persistent room‐temperature luminescence with lifetimes from seconds to days,^2^ organic materials have relatively short RTP lifetimes (typically <10 ms).^3^ To date, only a few examples of organic materials with persistent RTP (pRTP) in air with a long lifetime (>10 ms) have been described.^4^ Morantz et al.4a reported the first observation of weak RTP with lifetimes of several seconds in planar crystalline organic compounds, which was proposed to be related to dimer emission. In 2013, Adachi et al.4b, 5 reported a complex host–guest system to achieve pRTP in deuterated organic compounds. The inevitable phase separation in such a system, which results in unstable luminescence, limits its practical applications. Recently, our and other research groups have observed pRTP in crystals of different types of organic molecules, including 1,3,5‐triazines,4d phenylphosphines,4d ketones,4e,4g aldehydes,4f and sulfones.4c Various explanations for the rare phenomenon have been proposed, including the essential role of solvent molecules in the crystals,4e the chemical structure itself,4f strong coupling in H‐aggregated near‐planar molecules,4d and crystallization‐induced phosphorescence.4g There is currently no universal design strategy for new organic pRTP molecules. In addition, the limitation of low RTP quantum efficiency impedes their practical applications. Based on new results, we now propose a new mechanism for pRTP, which may also be applicable to the different molecules reported previously. We have identified the key role of intermolecular electronic coupling within the crystal structure of different subunits which possess different excited‐state configurations (i.e., nπ* and ππ* states). Based on this proposed mechanism, we have also designed two new organic molecules that achieve bright pRTP with the highest quantum efficiency reported to date. This may be a simple strategy to obtain efficient pRTP, which could be readily applied to diverse families of organic molecules.

As shown in Figure 1, the lowest triplet state (T_1_) excitons in organic molecules are generated by spin‐forbidden intersystem crossing (ISC) from the lowest singlet state (S_1_) excitons. Thus, heavy atoms and organic moieties with lone‐pair electrons can increase the ISC rate (*k*_ST_) through strong spin–orbit coupling to result in efficient phosphorescence.^6^ The heavy atoms are generally metallic ions and heavy halogen atoms and possess strong spin–orbit coupling capabilities. The organic moieties are usually aromatic aldehydes and ketones; thus, the lone–pair electrons contribute to an increase in *k*_ST_ through an allowed transition between nπ* and ππ* states (Figure 1 a, generally with a small energy gap). However, the radiative rate (*k*_r_) from the T_1_ state to the ground state (S_0_) also increases when the spin–orbit coupling is enhanced, thereby resulting in the short lifetime of phosphorescence (<10 ms, typically in the millisecond range, Table S1). By contrast, the spin–orbit coupling in typical hydrocarbon molecules occurs through a forbidden transition between two different ππ* states (generally with a large energy gap) and this transition is usually weak. Hence, they generally exhibit phosphorescence with long lifetimes because of the low *k*_r_ rate of the forbidden radiative process from T_1_ to S_0_ (Figure 1 b). Similarly, their phosphorescence quantum efficiencies are low, thus leading to weak emissions (Table S1).

**Figure 1:**
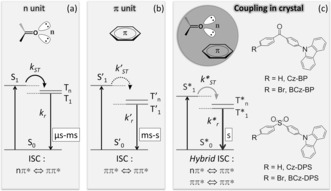
Energy level diagram of the relevant photophysical processes for the phosphorescence of organic molecules with a) nπ* excited state configuration (i.e., containing an n unit) and b) ππ* excited state configuration (i.e., containing a π unit). c) Proposed energy level diagram of the relevant photophysical processes for pRTP of coupled intermolecular n and π units in organic crystals, and examples of rationally designed molecular structures for pRTP utilizing the proposed mechanism. S_0_=ground state; S_1_=lowest singlet excited state; T_1_=lowest triplet excited state; T_*n*_=high‐level triplet excited state; ISC=intersystem crossing; *k*_ST_=ISC state; and *k_r_*=radiative rate. The superscripts (‘ and *) indicate excited states with different configurations.

Given the coupling structure of the exciplex state,^7^ we hypothesized that the intermolecular electronic coupling of units with different excited‐state configurations (i.e., nπ* and ππ*) involves both units in the photophysical processes, when their orbitals partially overlap (Figure 1 c). This coupling thus has hybrid ISC transitions with the advantages of improved *k*_ST_ and low *k*_r_ rates to promote bright pRTP. In addition, except for inert atmospheres and deuterated organic compounds, a rigid crystalline state was chosen to decrease the nonradiative decay (*k*_nr_) rates of the deactivation process by oxygen and heat, which can efficiently promote the RTP emissions.6b, 8, 9 Therefore, the intermolecular electronic coupling of subunits within a crystal may be a key factor for pRTP emission. A study of the single‐crystal structures of reported organic pRTP molecules reveals the presence of close intermolecular interactions of the n–π* unit (n unit, excited state with nπ* configuration) and π–π* unit (π unit, excited state with ππ* configuration) in most systems (for examples see Figure S1). We therefore propose a new explanation for organic pRTP: the intermolecular electronically coupled units (n and π units) combine their advantages, including high ISC and low radiative rates, in the photophysical processes of the crystal, which produces hybrid ISC transitions and leads to bright long‐lived RTP. With this idea in mind, we designed two kinds of twisted organic molecules containing a carbonyl or sulfonyl group as the n unit and a carbazolyl (Cz) group as the π unit (Figure 1 c).^10^, ^11^ The π unit also functions as an electron donor (D) and the n unit as an electron acceptor (A). Thus, similar to thermally activated delayed fluorescence (TADF) materials,^12^ the spatial overlap between the highest occupied molecular orbital (HOMO) and lowest unoccupied molecular orbital (LUMO) is reduced in D–A type twisted molecular structures. A small energy gap (Δ*E*_ST_) between the S_1_ and T_1_ excited states was thus obtained, which should increase the *k*_ST_ rate for phosphorescence. On the other hand, the n and π units have a tendency to form close intermolecular stacking in the crystals because of the electrostatic interactions between them. As expected, all the designed compounds show obvious pRTP emissions. To confirm our hypothesis, the pRTP properties of 4‐(9*H*‐carbazol‐9‐yl)benzophenone (Cz‐BP, Figure 1 c) were systematically investigated because of its simple ketone structure. When irradiated at 350 nm, the Cz‐BP crystalline powder exhibited dual emission, that is, both fluorescence and phosphorescence (Figure 2 a). The fluorescence peak at 431 nm with a shoulder at 453 nm showed a typical emission lifetime of 2.5 ns (Figure S2a in the Supporting Information). Two RTP peaks were observed at 570 and 624 nm. After the excitation lamp was turned off, the orange pRTP emission can be directly observed with the naked eye because of its ultralong luminescence lifetime of 0.49 s with a single‐exponential decay (Figure 2 a, and Figures S2b and S15).

**Figure 2:**
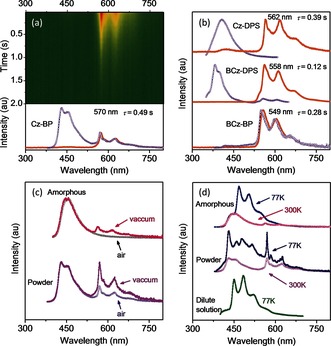
a) The transient photoluminescence (PL) decay image (delay 25 ms) of the Cz‐BP crystal powder sample, the color change from red to green indicates the decrease in emission intensity. Steady‐state PL spectra (open violet circles) and persistent phosphorescence spectra (delay 25 ms; filled orange circles) of different crystal powder samples: a) Cz‐BP and b) Cz‐DPS, BCz‐DPS, and BCz‐BP. Steady‐state PL spectra of the Cz‐BP samples under different conditions: c) in air or in vacuum and d) crystal powder, amorphous, and dilute solution in vacuum. The spectra and images were recorded in air at 300 K unless otherwise stated.

As expected, the ultralong‐lived RTP is due to the electronic coupling of intermolecular units in the Cz‐BP crystal (Figure 2 and Table S2). Firstly, the crystalline state is one of the essential factors to ensure the occurrence of pRTP emission by providing a rigid state insulated from oxygen in the air.^4^ This pRTP faded when the crystal powder was ground into an amorphous state or isolated in solutions, in which the increased intramolecular motion will quench the excitons. (Figure 2 c, Figures S3 and S4). The lack of RTP in the amorphous film in Figure 2 c is due to the presence of oxygen. Nevertheless, in a vacuum, pRTP is still observed in the amorphous sample because the intermolecular coupling between different Cz‐BP molecules can exist, even in an amorphous sample (Figure 2 c). The intensity of RTP in the crystalline powder also increased in vacuum because the sample still contained some amorphous parts. The pRTP was attributed to the intermolecular electronic coupling, which was further confirmed by comparing these results with the phosphorescence of a dilute solution of Cz‐BP at 77 K (Figure 2 d). The phosphorescence of a single Cz‐BP molecule was much bluer than the pRTP of the coupled structure, with peaks at 450, 484 and 521 nm. The luminescence lifetime was shorter at around 90 ms and also exhibited a single‐exponential decay. Such peaks were also observed in a similar range in the crystalline or amorphous samples at 77 K (Figure 2 d, and Figure S2c and Table S2). The intensity of such peaks, attributed to a single molecule, was lower than the emission of coupled molecules in the crystalline sample but much higher in the amorphous sample. These results indicated that most Cz‐BP molecules coupled and exhibit persistent phosphorescence in the crystalline state.

To gain further insight into the intermolecular electronic coupling mechanism, the single crystals of these compounds were studied by X‐ray structural analysis. The intermolecular interaction between the carbonyl (n) and Cz (π) units was observed in the crystals of Cz‐BP (Figure 3 a and Figure S5). The carbonyl group of the Cz‐BP molecule is stacked approximately parallel to the Cz group of a neighboring molecule. The distance between the two units is short (i.e., 3.373 Å for the oxygen atom and 3.561 Å for the carbon atom to the Cz plane), which results in significant intermolecular interactions between their orbitals. Thus, the coupling of typical n and π units in the crystal state could combine the excellent photophysical properties of both units, thereby resulting in intensive pRTP, as shown in Figure 1 c. An energy level diagram is shown in Figure 3 b using data estimated from the emission spectra of Cz‐BP (Figure S3). Both the energy levels of the singlet and triplet excited states of the coupled molecules (i.e., S′_1_ and T′_1_, respectively) decreased. Therefore, the T′_1_ state becomes the lowest‐energy state in the system, thus making the phosphorescence from the T′_1_ state possible and efficient.10b The coupled n and π units play critical roles in the processes of pRTP emission. These roles are supported by theoretical time‐dependent density functional theory (TD‐DFT) calculations on single molecules and coupled structures derived from single‐crystal structures (Figure 3, and Figures S9 and S11). For an isolated Cz‐BP molecule, there are only two main channels for ISC transition. However, the increased number of ISC channels (nine channels) enhances the ISC transition in coupled Cz‐BP molecules (Figure 3 c, d).^13^ Furthermore, significant intermolecular ISC channels are present in coupled Cz‐BP (such as, S_1_→T_7_ and T_8_, Figure 3 f).^14^

**Figure 3:**
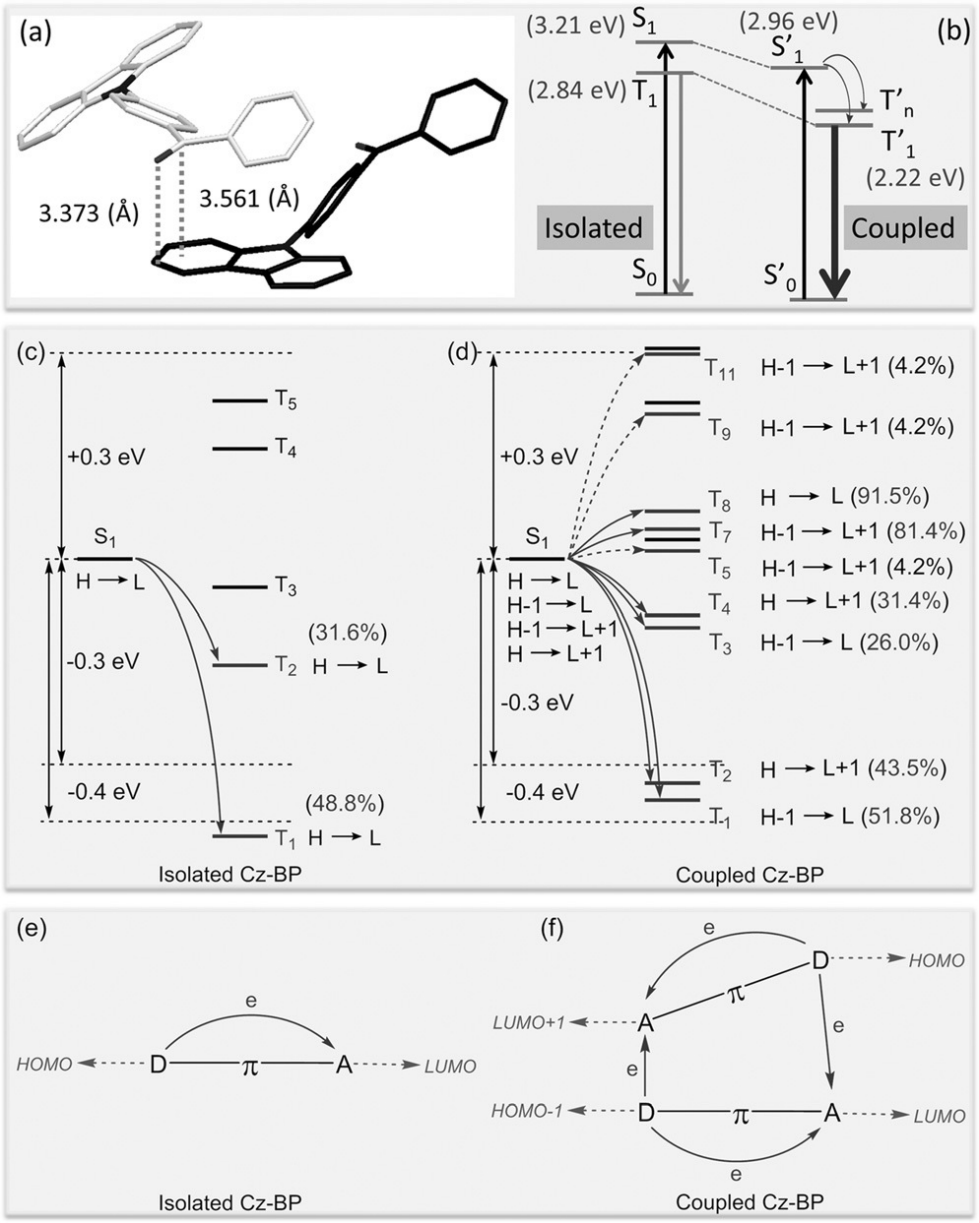
a) Intermolecular electronic coupling of the carbonyl and Cz groups in two Cz‐BP molecules that are in close proximity in a single crystal. b) Energy level diagram of the isolated and coupled Cz‐BP molecule(s). Schematic representations of the TD‐DFT calculated energy levels, main orbital configurations, and possible ISC channels of c) isolated Cz‐BP and d) coupled Cz‐BP at the singlet (S_1_) and triplet (T_*n*_) states. H and L refer to HOMO and LUMO, respectively. Schematic representations of the possible intramolecular and intermolecular ISC channels in e) isolated Cz‐BP and f) coupled Cz‐BP molecules. The carbonyl (electron acceptor) and the Cz (electron donor) units are labeled as A and D, respectively. The plain and dashed arrows refer to the major and minor ISC channels in (c), (d), (e), and (f), respectively.

It is clear that the intermolecular electronic coupling within the crystal structure of different n and π subunits has a key role in pRTP. The RTP of Cz‐BP exhibits a long lifetime; however, its absolute phosphorescence quantum efficiency (*Φ*_P_ , ca. 0.3 %) is low.4d As heavy halogen atoms facilitate strong spin–orbit coupling, their presence in the molecules should further improve the ISC rate, thereby promoting bright RTP emission. A bromine atom was, therefore, attached onto Cz‐BP at the *para* position with respect to the carbonyl group (BCz‐BP, Figure 1 c). As it is conjugated to the carbonyl group, the bromine atom will be involved in the ISC process and should further enhance the *k_ST_* rate of the n unit. The π units (Cz group) will keep its low radiative rates with little or no adverse effect from the bromine atom. Indeed, BCz‐BP exhibits markedly enhanced RTP emission with a high absolute *Φ*_P_ value of 5 % and a slightly decreased long lifetime of 0.28 s (Figure 2 b, and Figures S2b and S15). It is notable that this *Φ*_P_ value is more than twice the highest value reported to date (2.1 %).4d On the contrary, no pRTP will be observed in the bromide of Cz‐BP with bromine atom introduced into its π units.4g

Similar electronic coupling of intermolecular units was observed in the single crystal of BCz‐BP, where the distance between the n and π units of two neighboring molecules is even shorter (i.e., 2.983 Å for the oxygen atom and 3.524 Å for the carbon atom to the Cz plane; Figure 4 a and Figure S6). Therefore, the interaction between two coupled units in BCz‐BP crystals is stronger: this result is fully consistent with the fact that the pRTP emission is more intense for BCz‐BP compared to Cz‐BP. Interestingly, in the coupled BCz‐BP molecules, only intermolecular ISC transition is found based on the calculated results (Figures S9 and S12). That is, the intermolecular ISC transition could be dramatically increased by the enhancement of the spin–orbit coupling ability of the n unit, after introduction of a bromine atom. Thus, conjugating a heavy halogen atom into the n unit is a simple and efficient way to enhance the ISC rate of the intermolecular coupling of n and π moieties and lead to bright pRTP.

**Figure 4:**
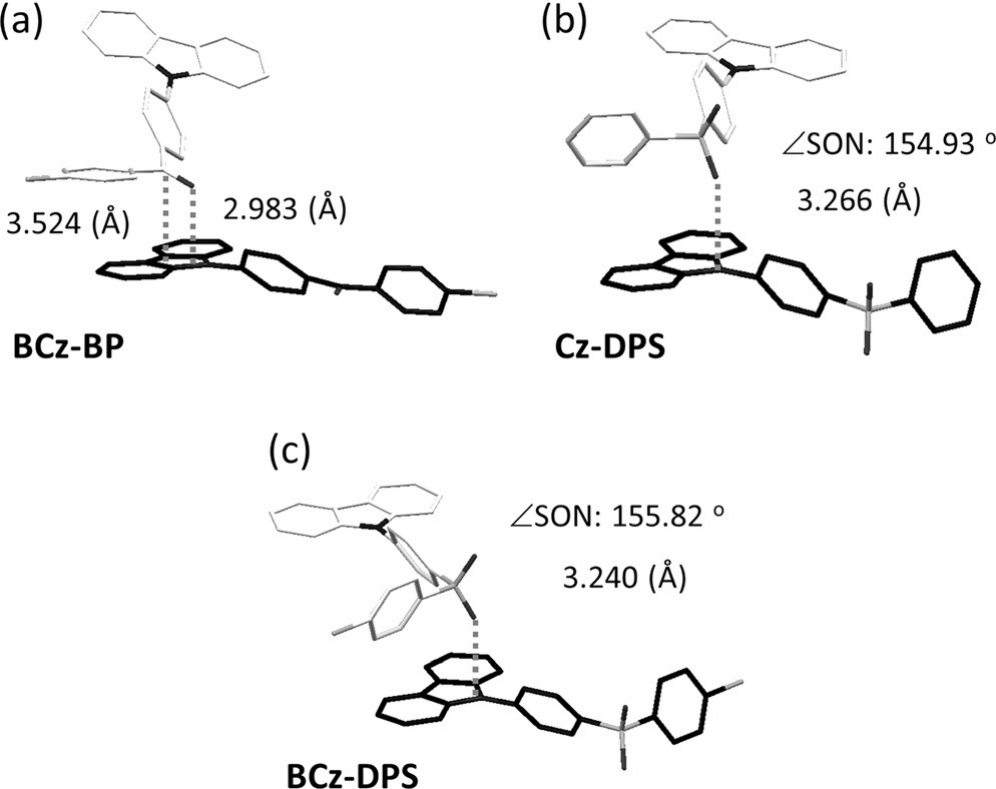
Intermolecular electronic coupling of the n and π units in two neighboring molecules in single crystal structures: a) Cz‐DPS, b) BCz‐BP, and c) BCz‐DPS. The distance from the n group to the coupled π plane Cz plane) is indicated by dashed lines: a) 2.983 Å (O‐Cz plane) and 3.524 Å (C‐Cz plane), b) 3.266 Å (O‐Cz plane), and c) 3.240 Å (O‐Cz plane). The relative angle (∡SON) between the S=O bond and the N atom of the Cz plane in (b) and (c) is also indicated: b) 154.93° and c) 155.82°.

When the n unit was changed to a sulfonyl group, our proposed mechanism was again supported by the experimental data. As shown in Figure 2 b, and Figures S2 d and S15, Cz‐DPS and BCz‐DPS exhibit obvious pRTP. Similar coupling of intermolecular n and π units was also observed in their single crystals (Figure 4 b, c and Figures S7 a and S8). All of these stacking motifs facilitate the orbital overlap between the n and the π units. The TD‐DFT calculations also show that newly generated intermolecular ISC channels in these coupled units promote RTP (Figure S10 and Figures S13 and S14). Therefore, as shown in Figure 1 c, the electronic coupling of n and π units in the crystals is the key factor for pRTP. This explanation should be applicable to different pRTP materials. The brominated sulfone derivative BCz‐DPS analogously exhibits much brighter RTP emission compared to Cz‐DPS (Figure S15). The absolute *Φ*_P_ value of BCz‐DPS is as high as 6 % (the highest reported value to date), and its lifetime is as long as 0.12 s.

Given that the pRTP of these molecules is apparent only in their crystalline state in the air, they are also unique mechanoluminochromic materials and are different from their inorganic analogues.^15^ Aside from the fluorescent emission color, the visible RTP of these materials is also mechanoresponsive, thus making them promising smart materials for use in dual‐responsive security protection with color‐coded and time‐resolved features (Figure 5 and Figure S16). As shown in Figure 5, the transient emission of the letter “π” (BCz‐BP) contain two parts: the light‐blue amorphous part and the yellow crystal part under 365 nm ultraviolet excitation. In terms of its time‐resolved feature, only the crystal part of letter “π” was clearly observed with the naked eye with an orange pRTP emission after the excitation lamp was turned off. When Cz‐BP is used, the amorphous part will gradually convert back to the crystal state when the sample was stored at room temperature for about 1 week, because of its low glass‐transition temperature (*T*_g_=33 °C) (Figures S16 and S17). This reversible process at room temperature without any other external stimuli makes Cz‐BP a promising simple dual‐responsive security protection material.

**Figure 5:**
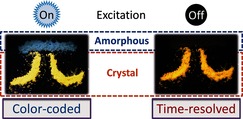
Dual‐responsive security protection applications involving the use of BCz‐BP for color‐coded and time‐resolved applications. When excited with 365 nm ultraviolet irradiation, the amorphous part (light blue) of security letter “π” was clearly distinguished from the crystal part (yellow). After the excitation is turned off, only the crystal part (orange) of the letter “π” can be observed.

In summary, we have presented a new explanation for pRTP in organic materials, based on a combined experimental and theoretical investigation. In such a mechanism, the n and π subunits that possess different excited‐state configurations electronically couple intermolecularly in the crystal, and thereby combine their individual advantages. Therefore, the crystals exhibit not only a high ISC rate derived from the n unit, but also a low radiative rate originating from the π unit, resulting in a hybrid ISC process to produce pRTP under ambient conditions. This mechanism is clearly distinct from those proposed recently, and it may also be applicable to these molecules reported previously. Consistent with this mechanism, we further demonstrate a molecular design strategy to obtain bright pRTP by simply incorporating a bromine atom in the conjugation of the n subunit to further increase the spin–orbit coupling ability of that unit. Bright long‐lived RTP with high phosphorescence quantum efficiency of 5 % and emission lifetime around 0.3 s was achieved for a pure organic compound. These results should promote new work on the rational design of new organic persistent RTP materials, including studies on intermolecular electronic coupling, This, in turn, may lead to the development of next‐generation organic luminescent materials with innovative applications in electronic and photonic technologies.

## Supporting information

As a service to our authors and readers, this journal provides supporting information supplied by the authors. Such materials are peer reviewed and may be re‐organized for online delivery, but are not copy‐edited or typeset. Technical support issues arising from supporting information (other than missing files) should be addressed to the authors.

Supplementary
