# Supplementary material for: Intermolecular Electronic Coupling of Organic Units for Efficient Persistent Room‐Temperature Phosphorescence
Source: Angew Chem Int Ed Engl. 2016 Jan 6;55(6):2181–5. doi: 10.1002/anie.201509224 (PMC5064736; doi:10.1002/anie.201509224)
Supplement: Supplementary file 1 — Supplementary [file ANIE-55-2181-s001.pdf]

Angewandte Chemie
Eine Zeitschrift der Gesellschaft Deutscher Chemiker

Supporting Information

Intermolecular Electronic Coupling of Organic Units for Efficient Persistent Room-Temperature Phosphorescence

Zhiyong Yang[+], Zhu Mao[+], Xuepeng Zhang, Depei Ou, Yingxiao Mu, Yi Zhang, Cunyuan Zhao, Siwei Liu, Zhenguo Chi,* Jiarui Xu, Yuan-Chun Wu, Po-Yen Lu, Alan Lien, and Martin R. Bryce**

anie_201509224_sm_miscellaneous_information.pdf

Contents

I. Synthesis and characterization. 1
II. Data for reference compounds 4
III. Photoluminescence spectra. 6
IV. Molecular stacking in crystals 9
V. Time-dependent density functional theory (TD-DFT) calculations. 13
VI. Photographs and other characterization. 22
VII. Corresponding color figures in the main text. 24
References 27

I. Synthesis and characterization.

Materials:

4-Bromobenzenesulfonyl chloride, benzensulfonyl chloride, 4-fluorobenzophenone, 4-fluoro-4'-bromo-benzophenone, fluorobenzene, carbazole and 2-methyltetrahydrofuran (2-Me-THF) were purchased from Aldrich. Potassium *tert*-butoxide was purchased from Alfa Aesar. The other chemicals were purchased from Aladdin Industrial Co. or Guangzhou Dongzheng Company (China). All materials were used as received without further purification. The compounds 4-(*N*-carbazolyl)benzophenone (**Cz-BP**), 4-bromo-4'-(*N*-carbazolyl)-benzophenone (**BCz-BP**), 4-(*N*-carbazolyl)-diphenylsulfone (**Cz-DPS**) and 4-bromo-4'-(*N*-carbazolyl)-diphenylsulfone (**BCz-DPS**) were prepared via previously reported experimental procedures.[1]

Characterization:

^{1}H and ^{13}C NMR spectra were obtained using a Bruker AVANCE 400 spectrometer in $CDCl_3$ or DMSO-d_6, with tetramethylsilane (TMS; $\delta = 0$) as the internal reference.

High resolution mass spectra (HRMS) were obtained using a Thermo MAT95XP spectrometer. Elemental analysis was conducted using an Elementar Vario EL analyzer. X-ray crystallographic intensity data were collected by using a Bruker Smart 1000 CCD diffractometer equipped with a graphite monochromated Enhance (Mo) X-ray source. The CCDC numbers of the compounds are 1402467-1402470. The UV−vis absorption spectra were obtained using a Hitachi U-3900 spectrophotometer. The photoluminescence (PL) spectra and time-resolved emission decay data were obtained using a spectrometer (FLSP980) from Edinburgh Instruments. The photoluminescence quantum yields were measured on a calibrated integrating sphere. The persistent phosphorescence spectra and transient PL decay images were obtained in delay of 25 ms from an Ocean-optical Maya 2000pro spectrometer. The WAXD measurements were obtained using a Rigaku X-ray diffractometer (D/max-2200) with an X-ray source of Cu Kα (λ= 0.15406 nm) at 40 kV and 30 mA, at a scan rate of 2° (2θ) per 1 min. The glass transition temperature of Cz-BP was evaluated via DSC on a NETZSCH DSC 204 F1 instrument in nitrogen at a heating rate of 10 °C/min.

Synthesis:

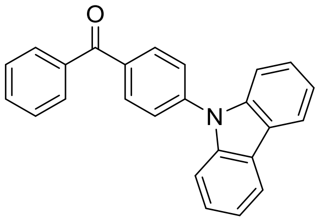

4-(*N*-carbazolyl)-benzophenone (Cz-BP) was prepared according to the similar experimental procedures reported previously,[1] only using 4-fluorobenzophenone instead of 1-fluoro-4-((4-iodophenyl)sulfonyl)benzene. ^{1}H NMR (400 MHz, $CDCl_3$): δ (ppm) 8.15 (d, *J* = 7.7 Hz, 2H), 8.08 (d, *J* = 8.3 Hz, 2H), 7.91 (d, *J* = 7.4 Hz, 2H), 7.74 (d, *J* = 7.4 Hz, 2H), 7.645 (t, *J* = 7.4 Hz, 1H), 7.57−7.52 (m, 4H), 7.45 (t, *J* = 7.4 Hz, 2H), 7.33 (t, *J* = 7.4 Hz, 2H); ^{13}C NMR (125 MHz, $CDCl_3$): δ (ppm) 195.6, 141.6, 140.3, 137.4, 136.0, 132.6, 131.9, 130.0, 128.4, 126.3, 126.2, 123.8, 120.6, 120.4, 109.8; HRMS, m/z: $[M]^+$ calcd. for $C_{25}H_{17}NO$, 347.1310; found, 347.1305; Anal.

calcd. for $C_{25}H_{17}NO$: C 86.43, H 4.93, N 4.03; found: C 86.21, H 4.72, N 3.91. Crystals for X-ray analysis were grown from a dichloromethane/hexane (1:3, v/v) mixture at room temperature.

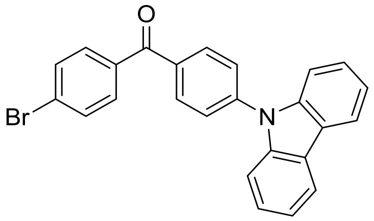

4-Bromo-4'-(*N*-carbazolyl)-benzophenone (BCz-BP) was prepared according to the similar experimental procedures reported previously,[1] only using 4-fluoro-4'-bromobenzophenone instead of 1-fluoro-4-((4-iodophenyl)sulfonyl) benzene. ^{1}H NMR (400 MHz, $CDCl_3$): δ (ppm) 8.16 (d, *J* = 7.5 Hz, 2H), 8.04 (d, *J* = 8.6 Hz, 2H), 7.78 (d, *J* = 8.6 Hz, 2H), 7.74 (d, *J* = 8.7 Hz, 2H), 7.69 (d, *J* = 8.7 Hz, 2H), 7.52 (d, *J* = 8.3 Hz, 2H), 7.44 (t, *J* = 8.3 Hz, 2H), 7.33 (t, *J* = 8.0 Hz, 2H); ^{13}C NMR (125 MHz, $CDCl_3$): δ (ppm) 194.5, 141.9, 140.2, 136.1, 135.5, 131.8, 131.5, 127.7, 126.4, 126.2, 123.9, 120.6, 120.5, 109.7; HRMS, m/z: $[M]^+$ calcd. for $C_{25}H_{16}BrNO$, 425.0415 and 427.0395; found, 425.0410 and 427.0389; Anal. calcd. for $C_{25}H_{16}BrNO$: C 70.44, H 3.78, N 3.27; found: C 70.25, H 3.53, N 3.46. Crystals for X-ray analysis were grown from a dichloromethane/hexane (1:3, v/v) mixture at room temperature.

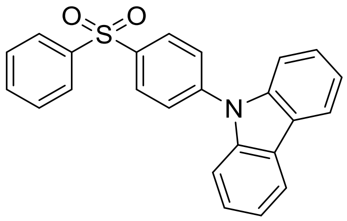

4-(*N*-carbazolyl)-diphenylsulfone (Cz-DPS) was prepared according to the similar experimental procedures reported previously,[1] only using 1-fluoro-4-(phenylsulfonyl)benzene instead of 1-fluoro-4-((4-iodophenyl) sulfonyl)benzene. ^{1}H NMR (400 MHz, $CDCl_3$): δ (ppm) 8.18 (d, *J* = 8.6 Hz, 2H), 8.13 (d, *J* = 7.8 Hz, 2H), 8.055 (d, *J* = 7.0 Hz, 2H), 7.75 (d, *J* = 8.6 Hz, 2H), 7.64 (t, *J* = 7.0 Hz, 1H), 7.58 (t, *J* = 7.0 Hz, 2H), 7.45−7.39 (m, 4H), 7.31 (t, *J* = 7.8 Hz, 2H); ^{13}C NMR (125 MHz, DMSO-d_6): δ (ppm) 142.4, 141.3, 139.9, 139.7, 133.4, 129.5,

129.4, 127.8, 127.0, 126.3, 123.9, 120.9, 120.5, 109.6; MS, m/z: $[M]^+$ calcd. for $C_{24}H_{17}NO_2S$, 383.0975; found, 383.0973; Anal. calcd. for $C_{24}H_{17}NO_2S$: C 75.17, H 4.47, N 3.65, S 8.36; found: C 75.23, H 4.58, N 3.44, S 8.19. Crystals for X-ray analysis were grown from a dichloromethane/hexane (1:1, v/v) mixture at room temperature.

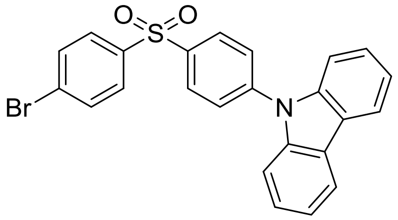

4-Bromo-4'-(*N*-carbazolyl)-diphenylsulfone (BCz-DPS) was prepared according to the similar experimental procedures reported previously,[1] only using 1-fluoro-4-((4-bromophenyl)sulfonyl)benzene instead of 1-fluoro-4-((4-iodophenyl) sulfonyl)benzene. ^{1}H NMR (400 MHz, $CDCl_3$): δ (ppm) 8.155 (d, *J* = 8.6 Hz, 2H), 8.13 (d, *J* = 7.7 Hz, 2H), 7.905 (d, *J* = 8.7 Hz, 2H), 7.765 (d, *J* = 8.7 Hz, 2H), 7.72 (d, *J* = 8.7 Hz, 2H), 7.44 (d, *J* = 7.7 Hz, 2H), 7.405 (t, *J* = 7.1 Hz, 2H), 7.32 (t, *J* = 7.1 Hz, 2H); ^{13}C NMR (125 MHz, DMSO-d_6): δ (ppm) 141.7, 140.1, 139.2, 138.4, 132.9, 129.5, 129.4, 128.0, 127.1, 126.4, 123.2, 120.8, 120.5, 109.7; MS, m/z: $[M]^+$ calcd. for $C_{24}H_{16}BrNO_2S$, 461.0080 and 463.0059; found, 461.0085 and 463.0062; Anal. calcd. for $C_{24}H_{16}BrNO_2S$: C 62.35, H 3.49, N 3.03, S 6.93; found: C 62.08, H 3.56, N 3.23, S 6.72. Crystals for X-ray analysis were grown from toluene at room temperature.

II. Data for reference compounds

Table S1. Photoluminescence parameters of organic molecules with different excited state configurations in dilute solution at low temperature (77 K).

Compounds		Phos. lifetime (s)	Quantum efficiency (%)		
			Phos.	ISC	Fluo.
ISC : $n\pi^* \Leftrightarrow \pi\pi^*$	Acetone	0.0006	4.2	–	–
	Acetophenone	0.0021	65	90	–
		0.0050	60	–	0
	Benzophenone	0.0060	84	–	0

	Benzaldehyde	0.0015	–	–	–
		0.0023	49	–	0
ISC : ππ* ⇔ ππ*	Benzene	4.5	15	–	17
		6.3	18	–	19
	Naphthalene	2.3	3.3	–	45
		2.6	3.9	29	45
	Anthracene	~0.04	0.03	–	34
	Pyrene	0.55	0.21	–	88
		0.58	0.22	22	92

[a]Data from reference [2]. For a molecule with data in two rows, the upper row is for a nonpolar solvent and the lower row for a polar solvent, respectively. Phos., phosphorescence; ISC, intersystem crossing; fluo., fluorescence. No data is indicated as "–".

As shown in Table S1, the intersystem crossing (ISC) process in organic molecules (containing lone-pair electrons) with an nπ* excited state configuration is through an allowed transition between nπ* state and ππ* states. When isolated in the solid solvents at low temperature, they usually exhibit short phosphorescence lifetimes (<10 ms) and high phosphorescence quantum efficiencies (≥ 50%, except acetone). On the contrary, the ISC process in typical hydrocarbon organic molecules with a ππ* excited state configuration is through the forbidden transition between two different ππ* states. Hence, such compounds generally exhibit phosphorescence with long lifetimes (>10 ms) and lower quantum efficiencies (< 10%, except benzene).

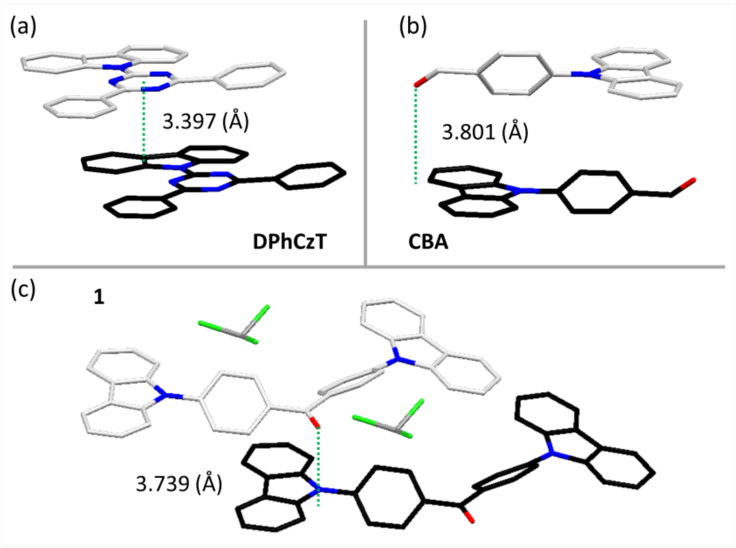

Figure S1. Intermolecular coupling of the n and π groups in two molecules that are adjacent in the single crystal of different compounds from literature references: (a) DPhCzT,[3] (b) CBA,[4] and (c) 4,4'-di(*N*-carbazolyl)benzophenone (**1**).[5] The distance from the n group to the coupled π plane (carbazolyl (Cz) plane) is indicated by green dashed lines: (a) 3.397 Å (centroid of 1,3,5-triazine group to Cz plane), (b) 3.801 Å (O−Cz plane), and (c) 3.739 Å (O−Cz plane).

As shown in Figure S1, close intermolecular interactions of the n–π* unit (n unit) and π–π* unit (π unit) are present in the single-crystal structures of these previously-reported organic persistent RTP molecules. This suggests our new explanation for organic persistent RTP may be generally applicable for a wide range of systems. The spectrum of 4-(9*H*-carbazol-9-yl)benzophone (Cz-BP) reported by Yuan *et al.*[6] is different from our result, however, similar with that of the first observation of weak persistent RTP in reference [7]. The Cz-BP molecules also exhibit a different stacking in the single-crystal, compared with our result. Nevertherless, its dimer stacking between two carbazolyl units is similar to that of the planar compounds in reference [7], indicating that their long-lived RTP may come from the same mechanism that is different from other organic persitent RTP compounds. As carbazole has a ground state dipole moment,[8] such a dipole moment maybe also exist in the stacked two carbazolyl units. Thus, such stacking can be considered as a donor−acceptor (D−A) pair to a certain extent, which could be one of the resons for their persistent RTP.

III. Photoluminescence spectra.

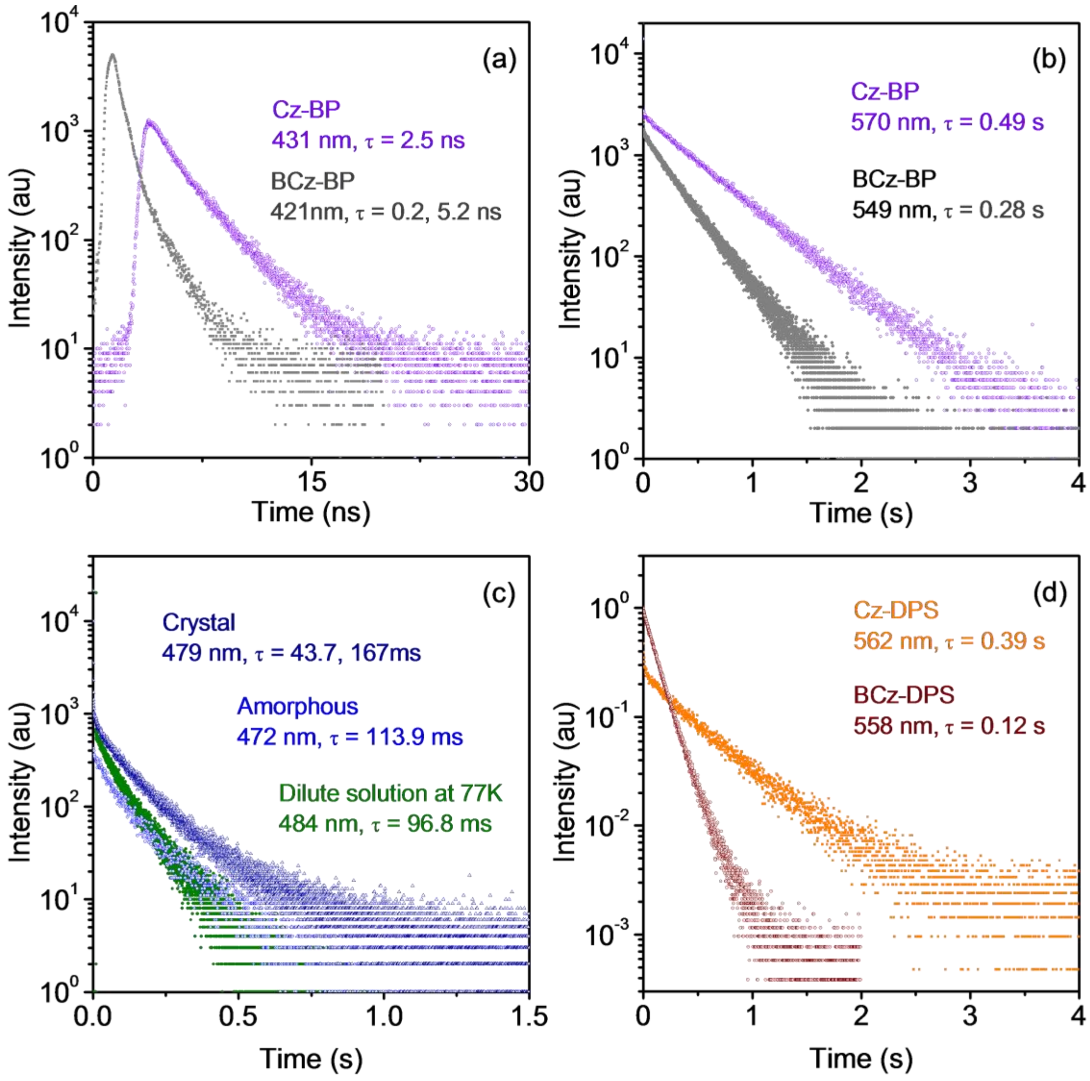

Figure S2. Time-resolved emission decay curves of different samples. (a) Fluorescence emissions of Cz-BP and BCz-BP in crystal powder samples. (b) Persistent RTP emissions of Cz-BP and BCz-BP in crystal powder samples. (c) Persistent RTP emissions of Cz-BP in crystal, amorphous, and dilute solution (at 77 K) samples, respectively. (d) Persistent RTP emissions of Cz-DPS and BCz-DPS in crystal powder samples. Luminescent peaks and lifetimes (τ) are indicated.

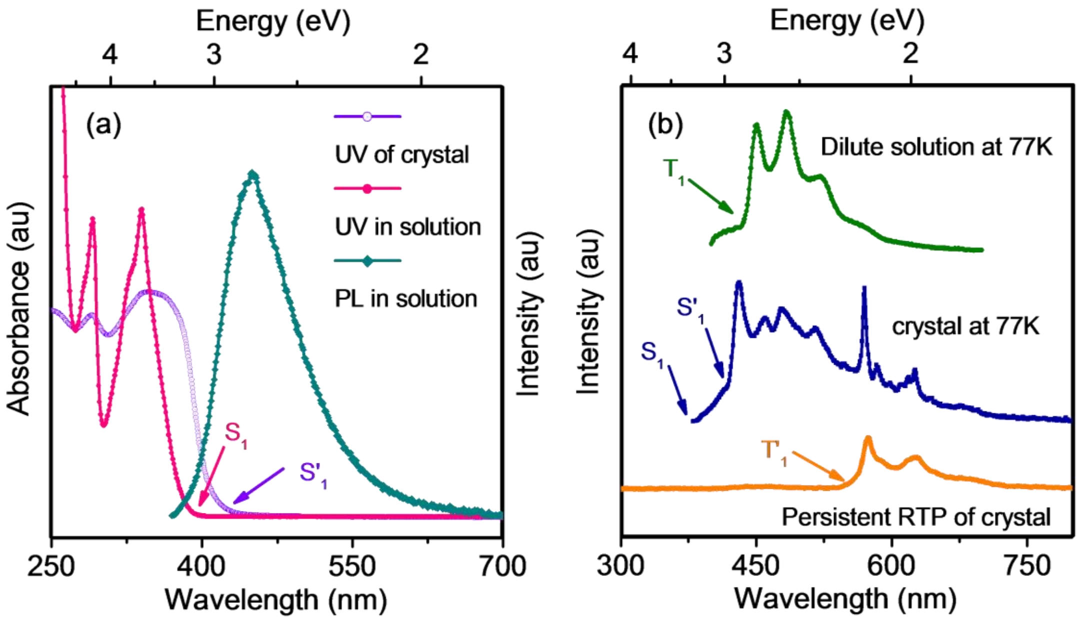

Figure S3. Determination of the energy levels of different excited states of Cz-BP. (a) UV−vis absorption spectra of Cz-BP in tetrahydrofuran (THF) solution (pink line) and solid state (crystal powder sample, violet line), PL spectra of Cz-BP in THF solution (dark cyan line). (b) Steady-state PL spectra of Cz-BP in 2-Me-THF solution (green line) and in solid state (crystal powder sample, blue line) at 77 K, persistent RTP spectrum of Cz-BP crystal powder (orange line) at room temperature. The onsets for determinations are indicated.

Table S2. Photoluminescence lifetimes (τ) of benzophenone and dibenzosulfone samples.

Compounds	Wavelength (nm)		Fluorescence[a]				Persistent phosphorescence[a]			
			τ_1 (ns)	A_1 (%)	τ_2 (ns)	A_2 (%)	τ_1 (ms)	A_1 (%)	τ_2 (ms)	A_2 (%)
Cz-BP Dilute solution	77 K	415	0.52	72.5	3.48	27.5				
		450					86.9	100	–	–
		484					96.8	100	–	–
	300 K	450	0.6	45.1	14.5	54.9				
Cz-BP Amorphous	77 K	445	3.1	57.2	6.3	42.8				
		472					114	100	–	–
	300 K	448	1.2	47.0	3.9	53.0				
		570					446	100	–	–
Cz-BP Crystal	77 K	479					43.7	26.6	167	73.4
		516					76.1	48.5	271	51.5
		570					548	100	–	–
		624					561	100	–	–
	300 K	431	2.5	100	–	–				
		570					479	100	–	–
		624					465	100	–	–
BCz-BP	300 K	421	0.3	72.6	1.6	27.4				
		549					287	100	–	–
		602					277	100	–	–
Cz-DPS	300 K	410	8.27	100	–	–				
		562					394	100	–	–
BCz-DPS	300 K	410	2.72	100	–	–				
		558					122	100	–	–

[a]Determined from the fitting function of $I_{(t)} = A_1 e^{-t/\tau_1} + A_2 e^{-t/\tau_2}$ according to the fluorescence and persistent RTP decay curves. All compounds are excited at 350 nm.

IV. Molecular stacking in crystals

Close stacking between carbonyl (or sulfonyl) and carbazolyl units (*i.e.*, n and π units) are found in single crystal X-ray structures of all the compounds. It is interesting that two stacking modes coexist in the single crystal of Cz-BP, including carbonyl stacks with carbazolyl group and two stacked carbazolyl units (Figure S5).

However, the distance (3.373 Å and 3.561 Å) between the carbonyl and carbazolyl groups is shorter than that of two stacked carbazolyl groups (3.653 Å). The ISC transition channels are found mainly between the carbonyl and carbazolyl group (*i.e.*, n and π units) from the TD-DFT calculated results (Figures 3, S9 and S11). The stacking between carbonyl and carbazolyl units thus plays the major role in the processes of persistent RTP of Cz-BP.

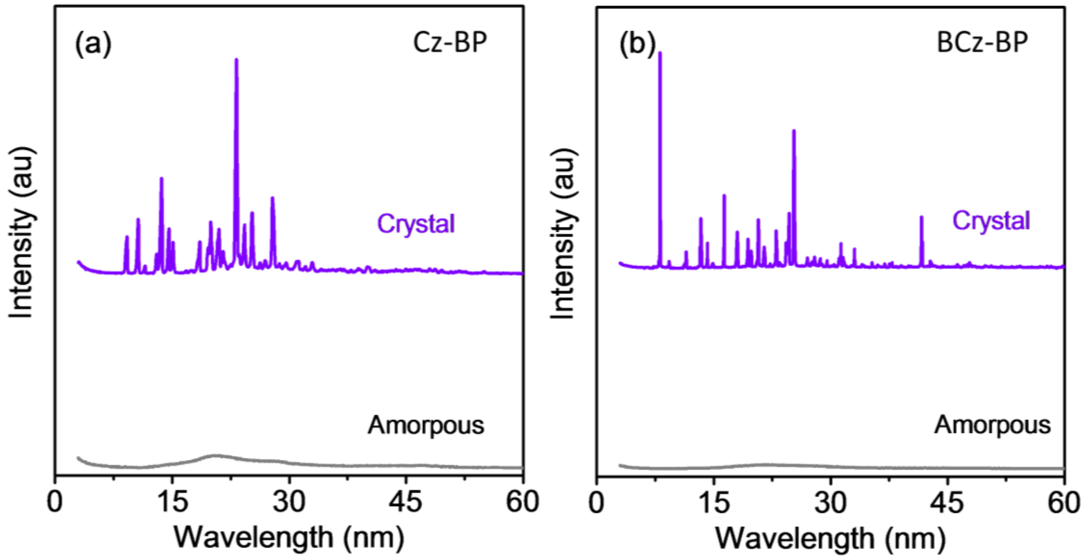

Figure S4. WAXD curves of different samples: (a) Cz-BP and (b) BCz-BP.

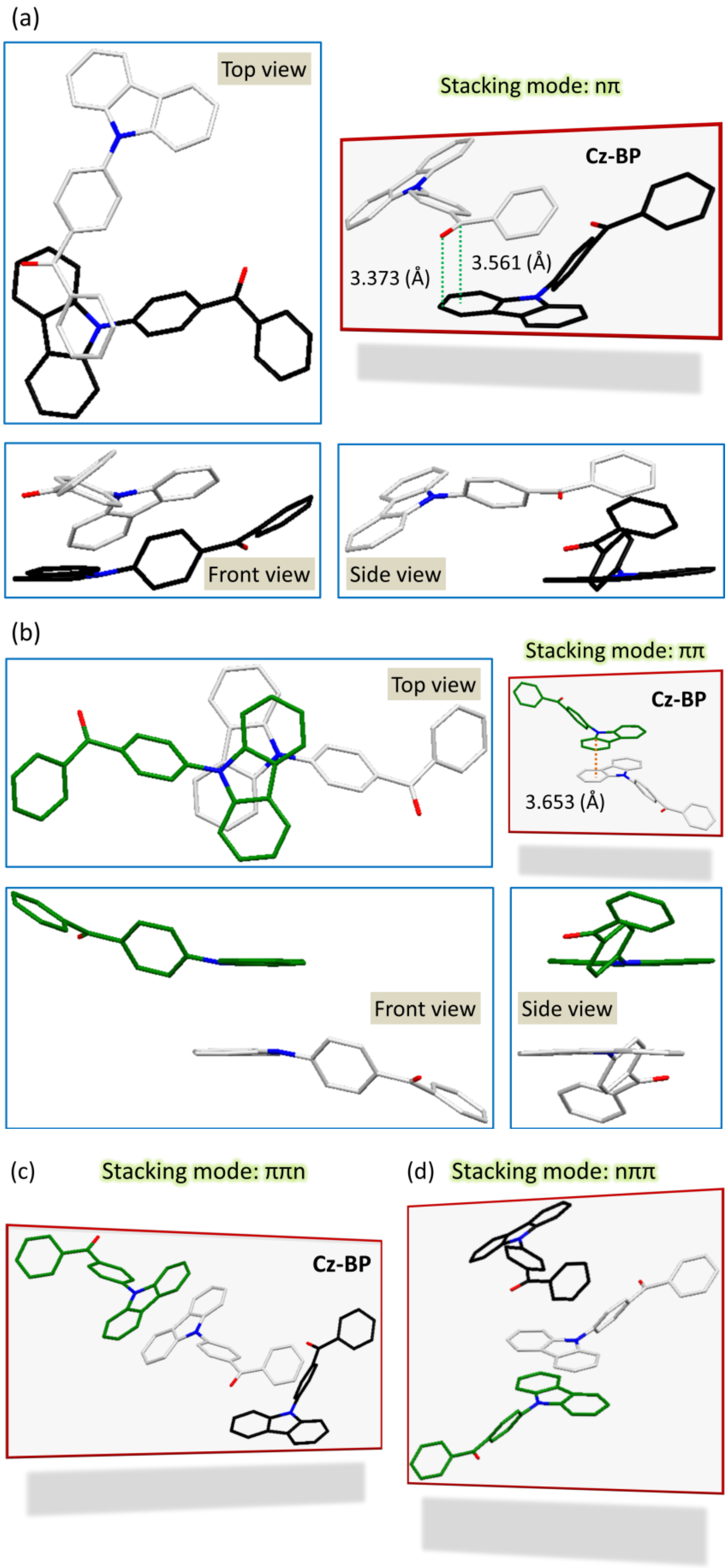

Figure S5. Molecular stackings in Cz-BP single-crystals. (a) The stacking between coupled carbonyl and carbazolyl units (n–π stacking mode), (b) the stacking between two carbazolyl units (π–π stacking mode), and two different modes of the above stackings present in the single crystal: (c) ππn stacking and (d) nππ stacking. The

distances between the carbonyl group and the coupled carbazolyl (Cz) plane are indicated by green dashed lines: 3.373 Å (O−Cz plane), 3.561 Å (C−Cz plane) in (a). The distance between two stacked carbazolyl groups is indicated by an orange dashed line: 3.653 Å (Cz plane−Cz plane) in (b).

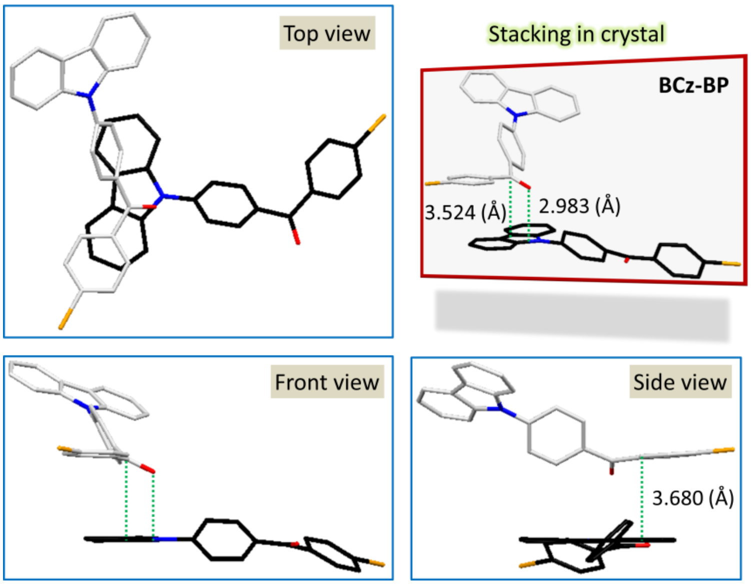

Figure S6. Molecular stacking in BCz-BP single-crystals. The distances from the carbonyl group and one carbon atom of a phenyl group to the coupled carbazolyl (Cz) plane are indicated by green dashed lines: 2.983 Å (O−Cz plane), 3.524 Å (C−Cz plane) and 3.680 Å (phenyl C−Cz plane).

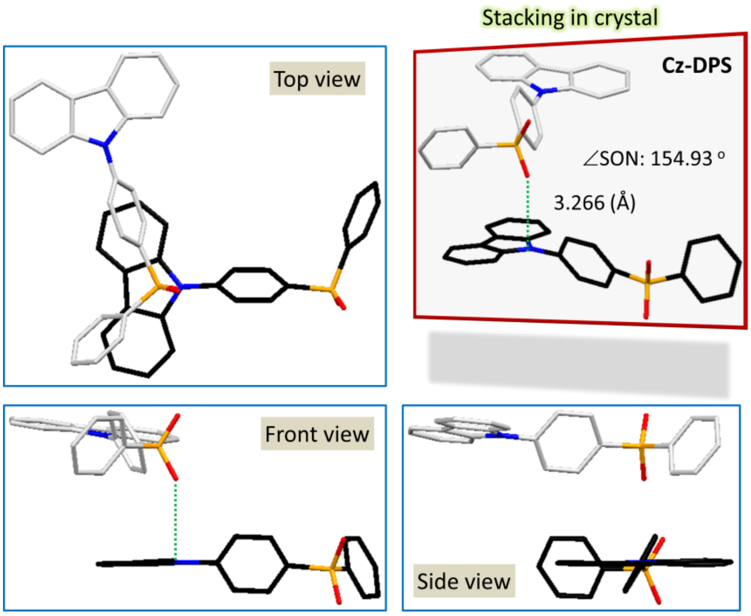

Figure S7. Molecular stacking in Cz-DPS single-crystals. The distances from one oxygen atom of a sulfonyl group to the coupled Cz plane is indicated by a green dashed line: 3.266 Å (O−Cz plane). The relative angle between the S=O double bond and the N atom of Cz plane is also shown: ∠SON = 154.93°.

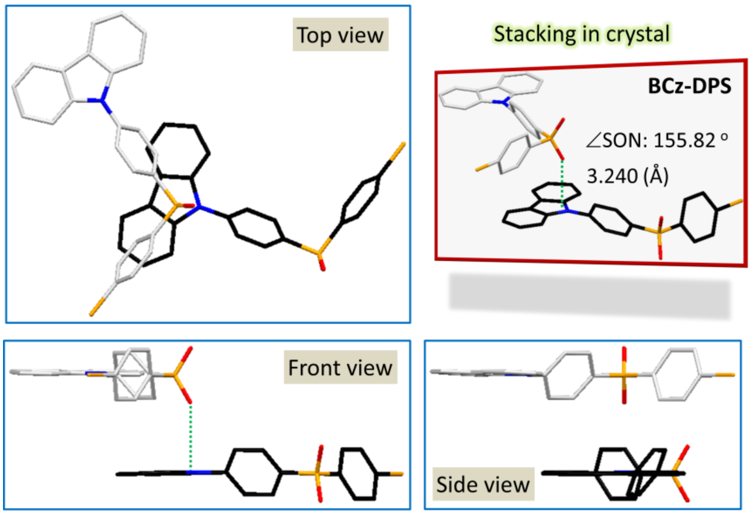

Figure S8. Molecular stacking in BCz-DPS single-crystals. The distances from one oxygen atom of a sulfonyl group to the coupled Cz plane is indicated by a green dashed line: 3.240 Å (O−Cz plane). The relative angle between the S=O double bond and the N atom of Cz plane is also shown: ∠SON = 155.82°.

V. Time-dependent density functional theory (TD-DFT) calculations.

The Gaussian 09[9] program was utilized to perform the TD-DFT calculations. The ground state (S_0) geometries were obtained from the single crystal structures and no further geometry optimization was conducted in order to maintain the specific molecular configurations and corresponding intermolecular locations. The exciton energies of the *n*-th singlet (S_n) and *n*-th triplet states were obtained on the corresponding ground state structure using the combination of TD[10]-B3LYP[11]/6-31G* (6-311G* for Br atoms). Kohn-Sham frontier orbital analysis and spin density distributions were obtained in order to elucidate the mechanisms of possible singlet-triplet intersystem crossings (ISC). The possible S_1 to T_n ISC channels are

believed to share part of the same transition orbital compositions, and the energy levels of T_n are considered to lie within the range of E_{S1} ± 0.3 or 0.4 eV. Especially, the major ISC channels are mainly determined based on two elements. Firstly, the ratio of the same transition configuration in S_1 and T_n should be large in all the transition orbital compositions. Secondly, the energy gap between S_1 and the specific T_n state should be small. When the energy of T_n is lower than S_1, the first element is considered to be more important. The determination of minor ISC channels is vice versa. In the following schematic representations of Figures S11, S12, S13, and S14, the notations H and L refer to the highest occupied molecular orbital (HOMO) and the lowest unoccupied molecular orbital (LUMO), respectively. The plain arrows and dashed arrows refer to the major and minor ISC channels, respectively. The carbazolyl group (electron donor) is referred to as D while the carbonyl group (electron acceptor) is referred to as A. The phenyl bridge is located between the D and A moieties, and thus the molecules are represented as D-π-A.

From the calculations it can be concluded that both the coupled molecules are n–π*/π–π* transition hybrid structures during the ISC processes. All of the ISC transitions are from the Cz sub-units to the carbonyl groups. After coupling in the crystals, significant intermolecular ISC channels are found. For BCz-BP, there are only intermolecular ISC channels in the coupled molecules (Figure S12). For BCz-DPS, all ISC channels in the coupled molecules contain intermolecular component (Figure S14). For Cz-BP, in addition to newly generated intermolecular ISC transition channels, the number of ISC channels increased in the coupled molecules (Figure 3 and S11). For Cz-DPS, there are newly generated intermolecular ISC transition channels through S_2 state. Thus, the coupling of carbonyl and carbazolyl groups (i.e., n and π groups) is the main reason for the enhanced ISC transitions for triplet excitons, leading to persistent RTP (Figure S13). The persistent RTP of these compounds become brighter with the increase and enhancement of such intermolecular ISC channels: BCz-DPS ≈ BCz-BP > Cz-BP > Cz-DPS.

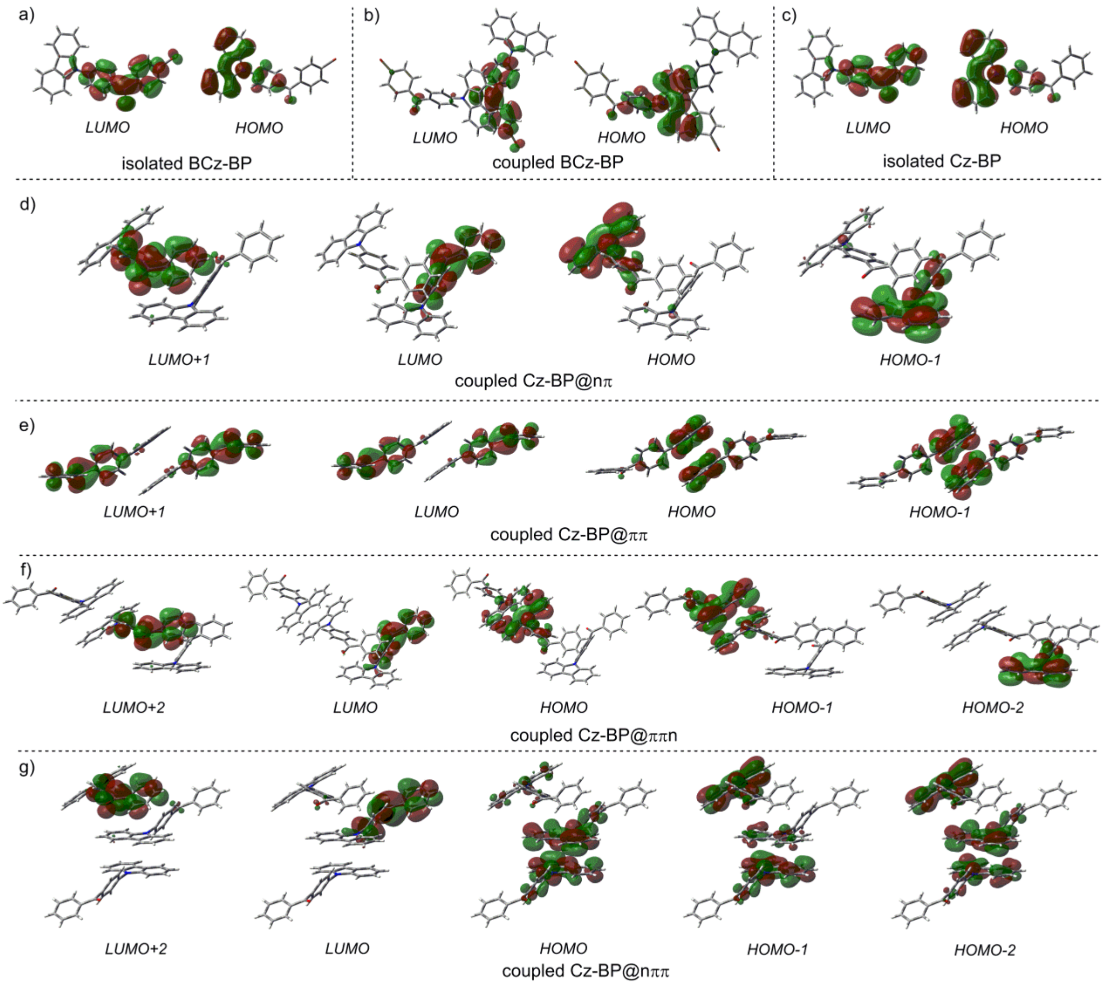

Figure S9. Depicted are the Kohn-Sham frontier orbitals (isovalue is 0.02) obtained from the (a) isolated BCz-BP, (b) coupled BCz-BP, (c) isolated Cz-BP, (d) coupled Cz-BP@nπ, (e) coupled Cz-BP@ππ, (f) coupled Cz-BP@ππn, and (g) coupled Cz-BP@nππ.

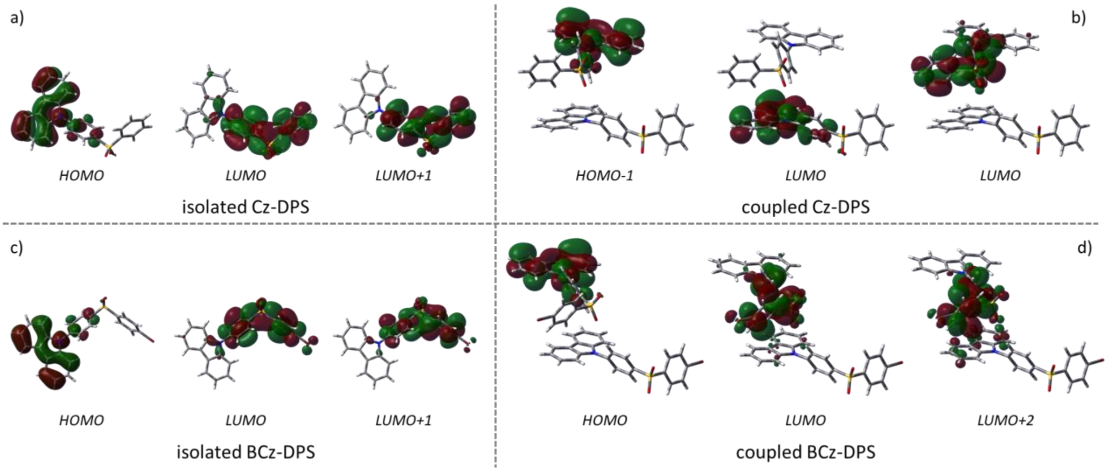

Figure S10. Depicted are the Kohn-Sham frontier orbitals (isovalue is 0.02) obtained from the (a) isolated Cz-DPS, (b) coupled Cz-DPS, (c) isolated BCz-DPS, (d) coupled BCz-DPS.

As is clearly shown in Figure S9 and S10, as expected, the HOMO and HOMO-n orbitals are primarily located at the carbazolyl moieties, while the LUMO and LUMO+n orbitals are found at the carbonyl moieties. Interestingly, for coupled BCz-DPS, the LUMO orbital partially extends to the carbazolyl group of the close coupled molecule, and it becomes more obvious for the LUMO+2 orbital. This indicates that the transitions involving both LUMO and LUMO+2 orbitals contain an *inter*molecular component.

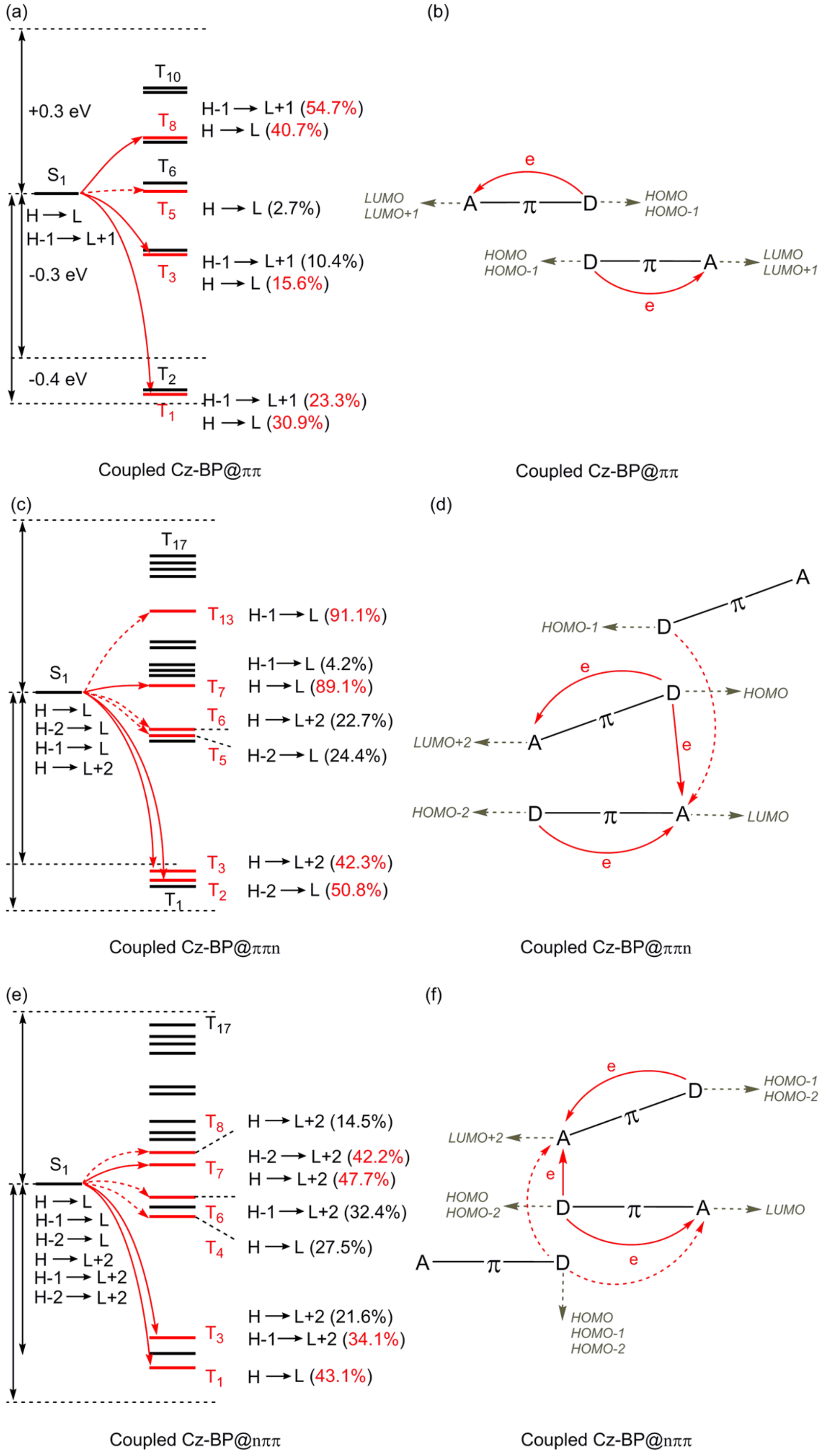

Figure S11. Schematic representations of the TD-DFT calculated energy levels, main orbital configurations and possible ISC channels of (a) coupled Cz-BP@ππ

(according to the ππ stacking shown in Figure S5b), (c) coupled Cz-BP@ππn (according to the ππn stacking shown in Figure S5c) and (e) coupled Cz-BP@nππ (according to the nππ stacking shown in Figure S5d) at the singlet (S_1) and triplet (T_n) states. The schematic representations in (b) coupled Cz-BP@ππ, (d) coupled Cz-BP@ππn and (f) coupled Cz-BP@nππ are the relative possible intramolecular and intermolecular ISC channels.

As can be seen above, and also in Figure 3c-f, all of the ISC transitions are from the Cz groups to the carbonyl groups. In an isolated Cz-BP molecule (Figure 3e), intramolecular ISC processes are exclusive. However, significant intermolecular ISC channels are found in coupled Cz-BP (Figure 3f, S11d and S11f). For coupled Cz-BP@nπ, coupled Cz-BP@ππn and coupled Cz-BP@nππ there are similar intermolecular ISC channels. In contrast, in coupled Cz-BP@ππ, only the intramolecular ISC channels are observed (Figure S11b). These results reveal that the stacks between Cz groups (π unit) play minor roles in the intermolecular ISC transitions of the system. These results also indicate, as above, that the coupling of carbonyl and Cz groups (*i.e.*, n and π units) in Cz-BP molecules is the main reason for enhanced ISC transition for triplet excitons, which will lead to persistent RTP.

Additionally, as all the major ISC transition channels in the systems of coupled Cz-BP@ππn and coupled Cz-BP@nππ can be found in the coupled Cz-BP@nπ system, the stacks between Cz groups (π unit) in the two former structures can be ignored. Thus, the result of a coupled Cz-BP@nπ system can be used as the representative one for coupled Cz-BP molecules.

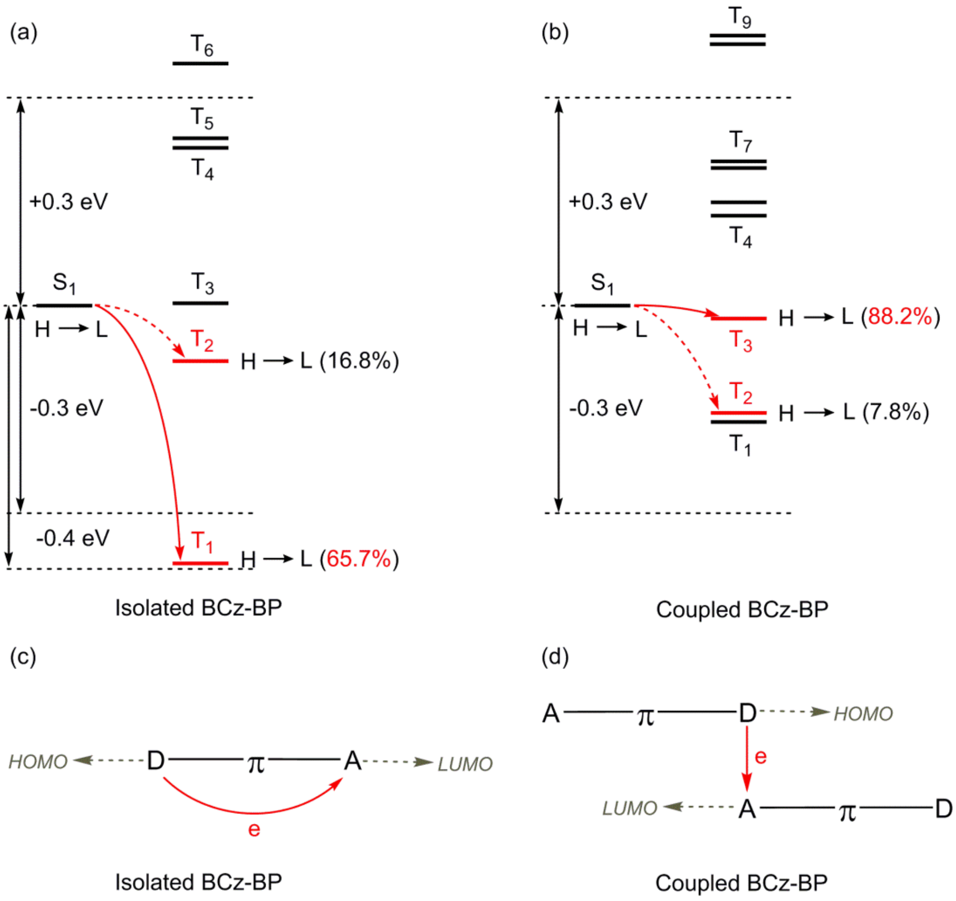

Figure S12. Schematic representations of the TD-DFT calculated energy levels, main orbital configurations and possible ISC channels of (a) isolated BCz-BP and (b) coupled BCz-BP at the singlet (S_1) and triplet (T_n) states. The schematic representations in (c) isolated BCz-BP and (d) coupled BCz-BP are the relative possible intramolecular and intermolecular ISC channels.

As can be seen in Figure S12, all of the ISC transitions are from the Cz groups to the carbonyl groups. In an isolated BCz-BP molecule (Figure S12c), *intra*molecular ISC processes are exclusive. However, only *inter*molecular ISC channels are found in coupled BCz-BP (Figure S12d). These results indicate that the coupling of carbonyl and carbazolyl groups (*i.e.*, n and π groups) in BCz-BP molecules is the main reason for the enhanced ISC transition for triplet excitons, which will lead to persistent RTP.

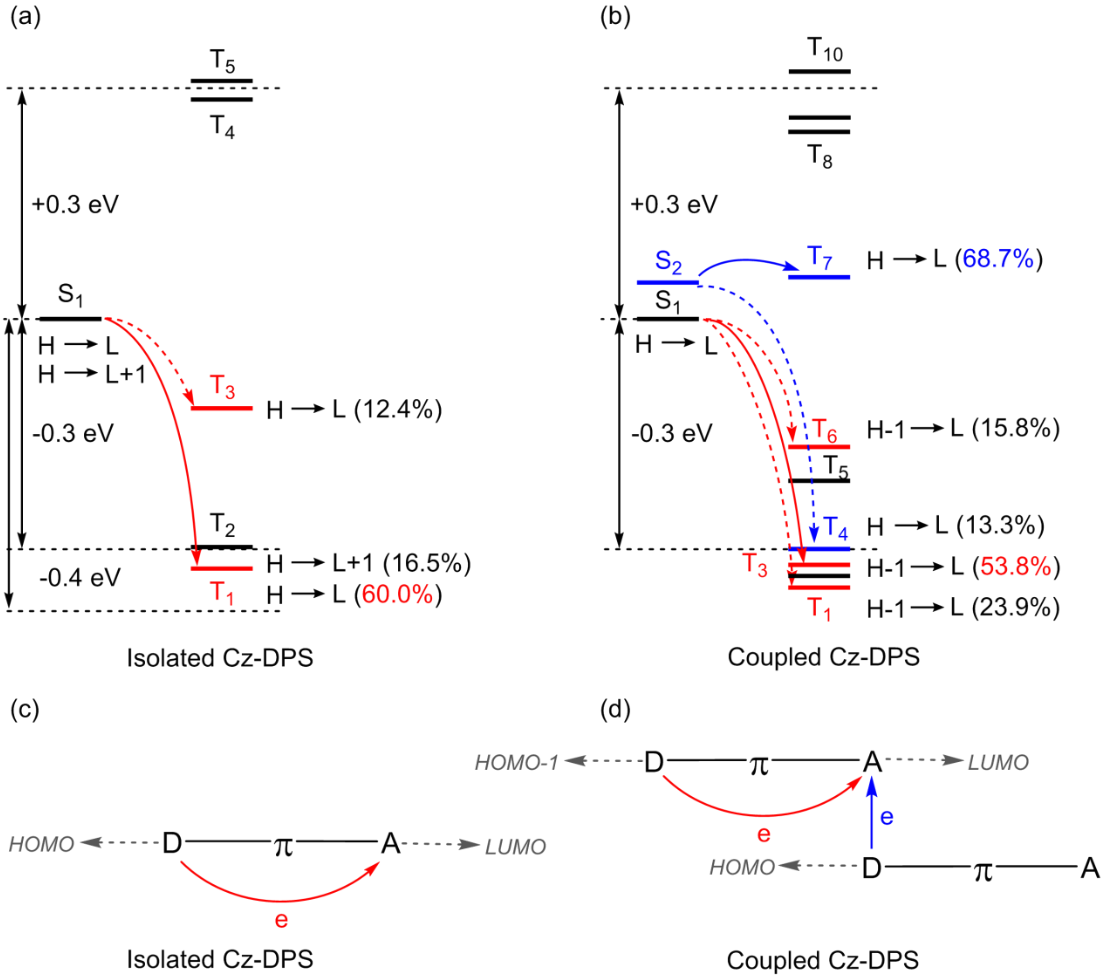

Figure S13. Schematic representations of the TD-DFT calculated energy levels, main orbital configurations and possible ISC channels of (a) isolated Cz-DPS and (b) coupled Cz-DPS at the singlet (S_1 and S_2) and triplet (T_n) states. The schematic representations in (c) isolated Cz-DPS and (d) coupled Cz-DPS are the relative possible intramolecular and intermolecular ISC channels. The red arrows and blue arrows refer to the ISC channels from S_1 and S_2, respectively.

As can be seen in Figure S13, all of the ISC transitions are from the Cz groups to the carbonyl groups. In an isolated Cz-DPS molecule (Figure S13c), *intra*molecular ISC processes are exclusive. However, *inter*molecular ISC channels are found in coupled Cz-DPS through S_2 to T_7 and S_2 to T_7 (Figure S13d). These results indicate that the coupling of carbonyl and carbazolyl groups (*i.e.*, n and π groups) in Cz-DPS

molecules is the main reason for the enhanced ISC transition for triplet excitons, which will lead to persistent RTP.

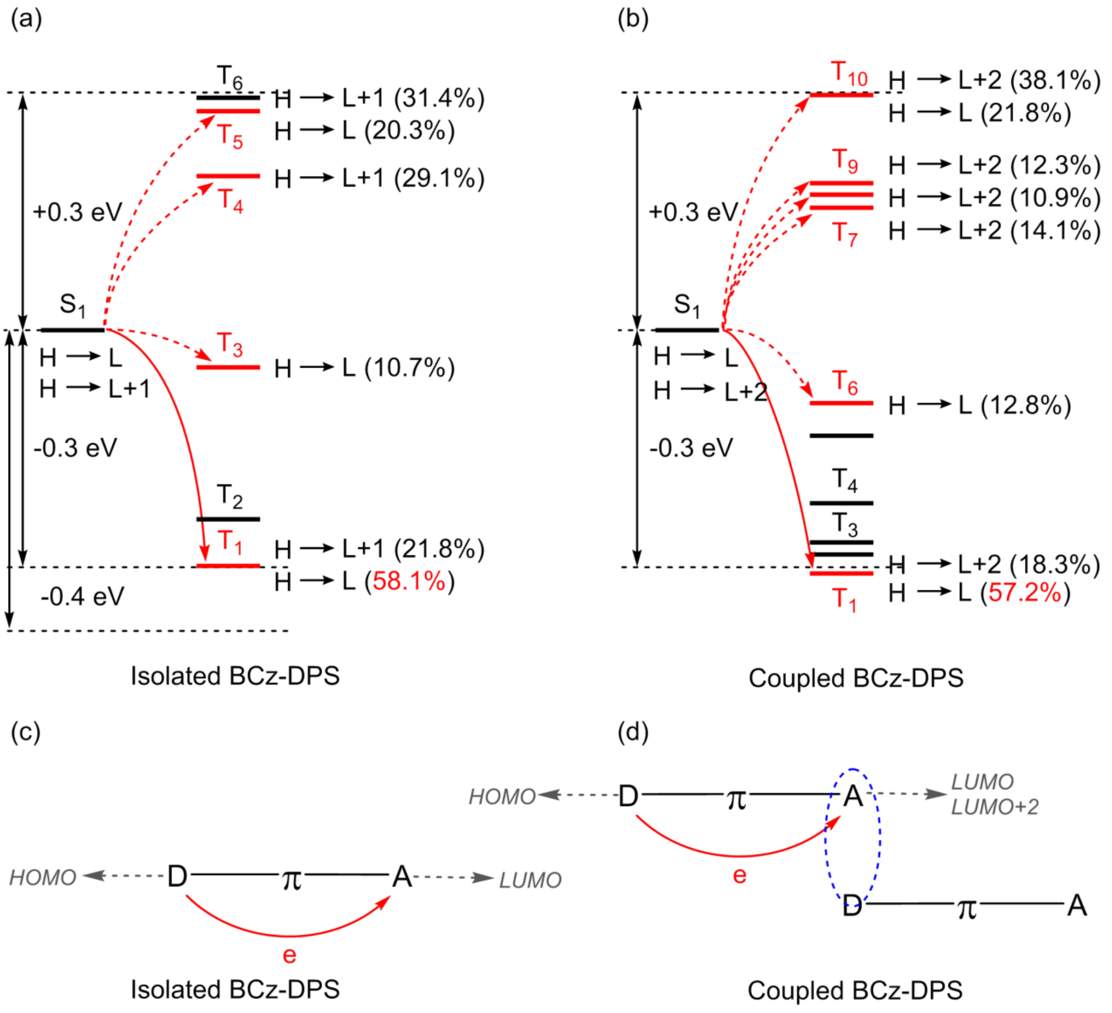

Figure S14. Schematic representations of the TD-DFT calculated energy levels, main orbital configurations and possible ISC channels of (a) isolated BCz-DPS and (b) coupled BCz-DPS at the singlet (S_1) and triplet (T_n) states. The schematic representations in (c) isolated BCz-DPS and (d) coupled BCz-DPS are the relative possible intramolecular and intermolecular ISC channels. Schematic drawing of the orbital scope of LUMO and LUMO+2 in coupled BCz-DPS is shown as blue circle (Figure S10d).

As can be seen in Figure S14, all of the ISC transitions are from the Cz groups to the carbonyl groups. In an isolated BCz-DPS molecule (Figure S14c), *intra*molecular

ISC processes are exclusive. In spite of no *inter*molecular ISC channels are found in coupled BCz-DPS (Figure S14d), however, as the LUMO and LUMO+2 orbitals of coupled BCz-DPS contain *inter*molecular component (Figure S10d), all of these transitions involved these orbitals actually intermolecular transitions. These results indicate that the coupling of carbonyl and carbazolyl groups (*i.e.*, n and π groups) in BCz-DPS molecules is the main reason for the enhanced ISC transition for triplet excitons, which will lead to persistent RTP.

VI. Photographs and other characterization.

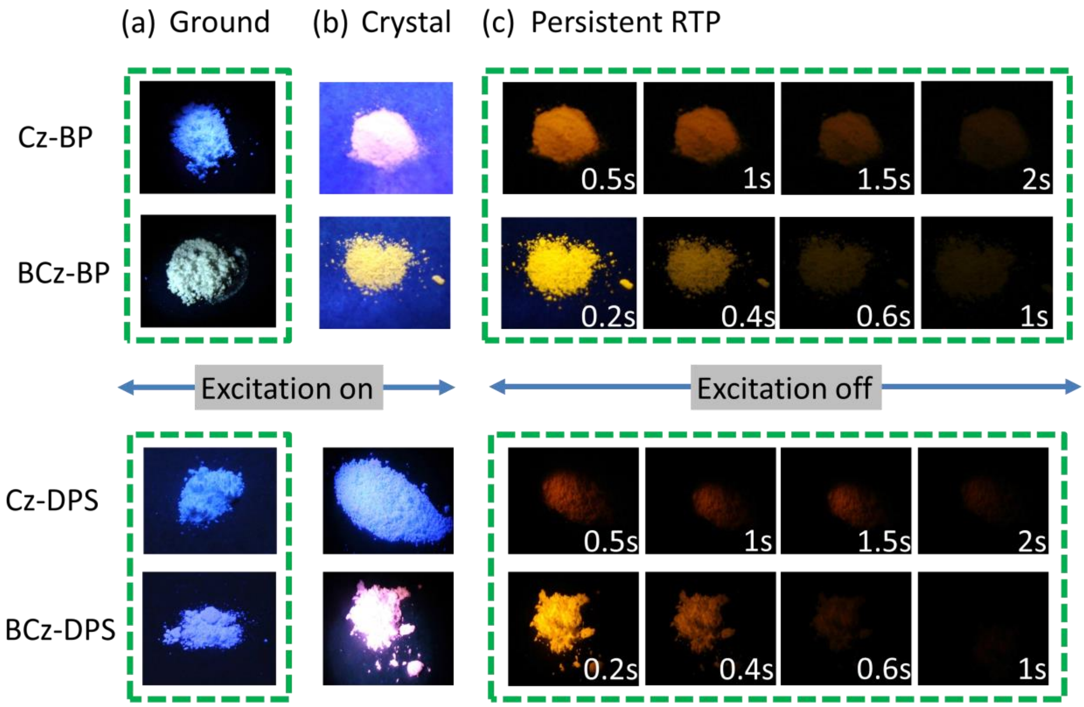

Figure S15. The photographs of samples taken under different conditions: (a) and (b) emission when excitation with an ultraviolet lamp (365 nm) turned on; (c) persistent RTP after excitation turned off; the time intervals are indicated.

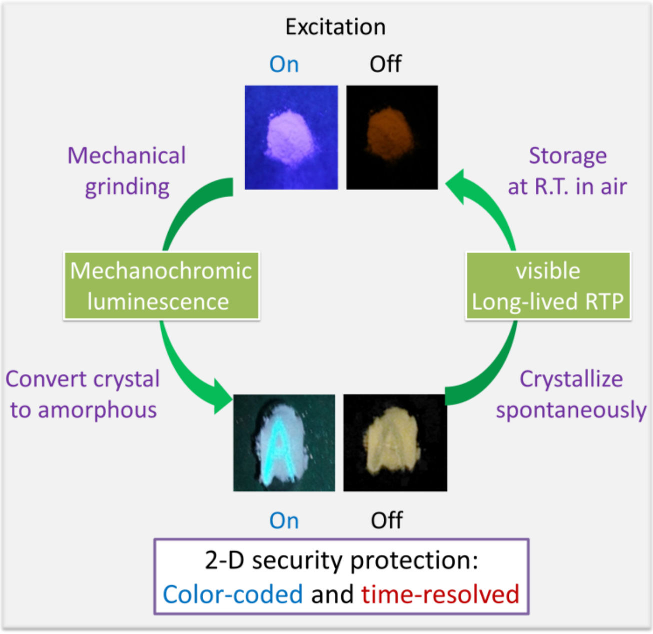

Figure S16. Two-dimensional security protection applications involving the use of Cz-BP for color-coded and time-resolved data. When excited with 365 nm ultraviolet irradiation, the ground security letter 'A' was clearly recognizable. After the excitation is turned off, the non-emissive letter 'A' is observed in the persistent RTP background. The letter 'A' was etched on the crystalline powder via gentle grinding, the ground area was converted to an amorphous state and subsequently quenched the persistent RTP. The letter then gradually disappeared when the sample was stored at room temperature in the air for about 1 week because the Cz-BP molecules underwent spontaneous crystallization as a result of the compound's low glass transition temperature (T_g = 33 °C) (Figure S17).

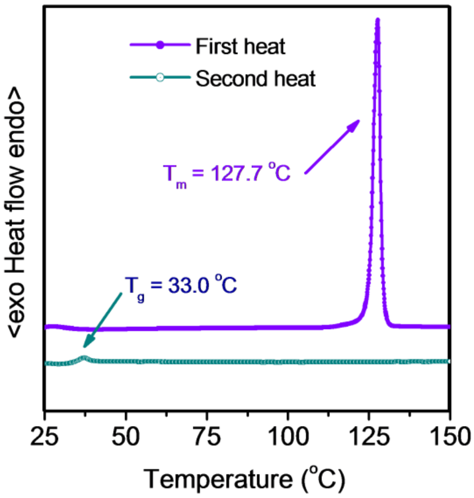

Figure S17. DSC curves of Cz-BP crystal powder sample. The glass transition temperature (T_g) and melting temperature (T_m) are indicated.

VII. Corresponding color figures in the main text.

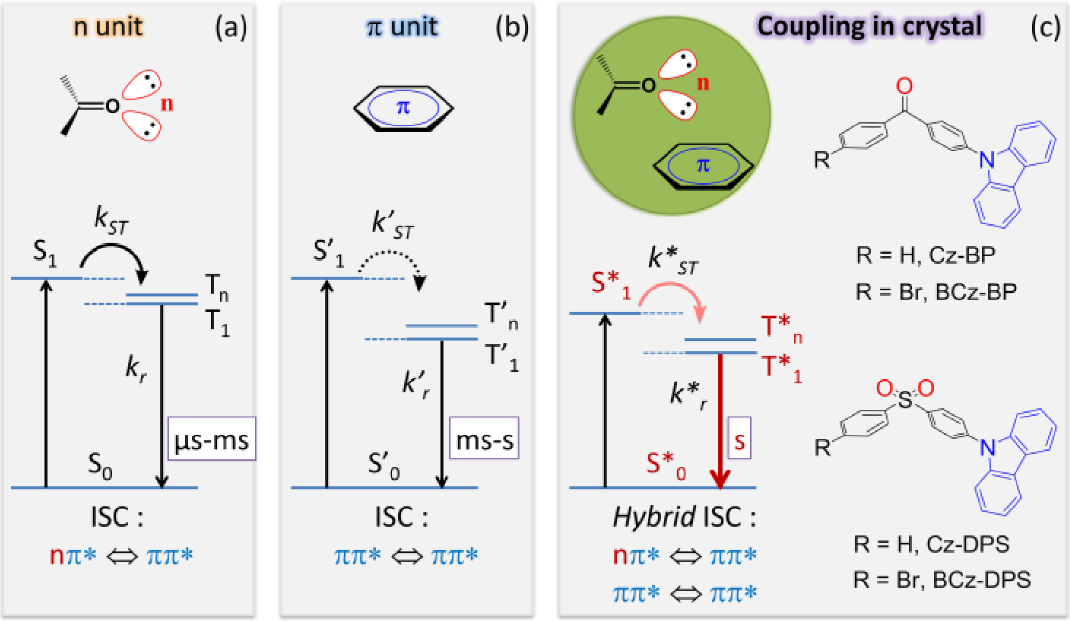

Figure 1. Energy level diagram of the relevant photophysical processes for the phosphorescence of organic molecules with (a) nπ* excited state configuration (i.e., containing n unit) and (b) ππ* excited state configuration (i.e., containing π unit). (c) Proposed energy level diagram of the relevant photophysical processes for pRTP of coupled intermolecular n and π units in organic crystals, and examples of rationally designed molecular structures for pRTP utilizing the proposed mechanism. S_0, the ground state; S_1, the lowest singlet excited state; T_1, the lowest triplet excited state; T_n, the high-level triplet excited state; ISC, intersystem crossing; k_{ST}, the ISC state; and k_r, the radiative rate. The superscript indicates the excited state with different configurations.

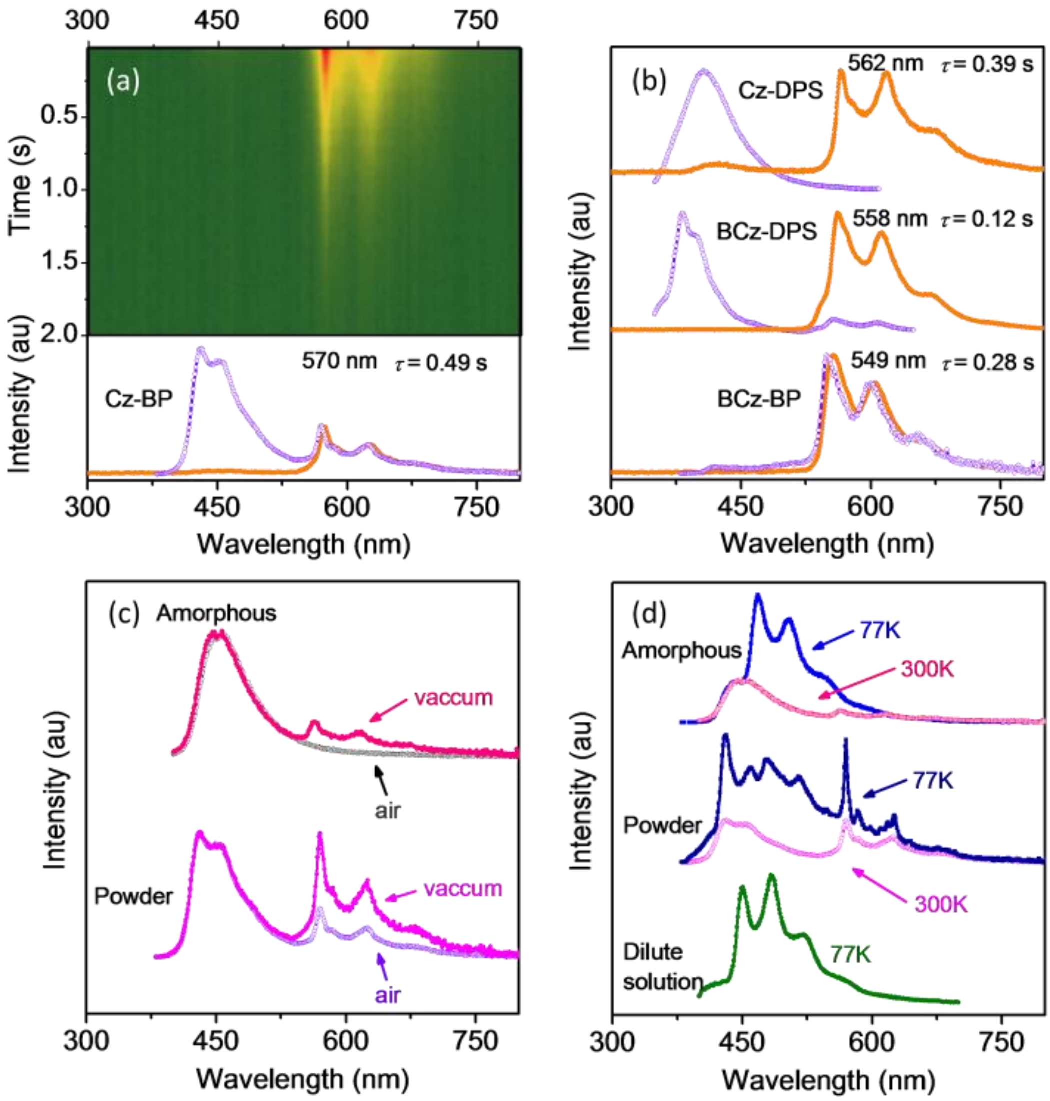

Figure 2. (a) The transient photoluminescence (PL) decay image (delay 25 ms) of the Cz-BP crystal powder sample, the color change from red to green indicates the decrease in emission intensity. Steady-state PL spectra (violet circle) and persistent phosphorescence spectra (delay 25 ms, orange ball) of different crystal powder samples: (a) Cz-BP and (b) Cz-DPS, BCz-DPS, and BCz-BP. Steady-state PL spectra of the Cz-BP samples under different conditions: (c) in air or in vacuum and (d) crystal powder, amorphous and dilute solution in vacuum. The spectra and images were recorded in air at 300 K unless otherwise stated.

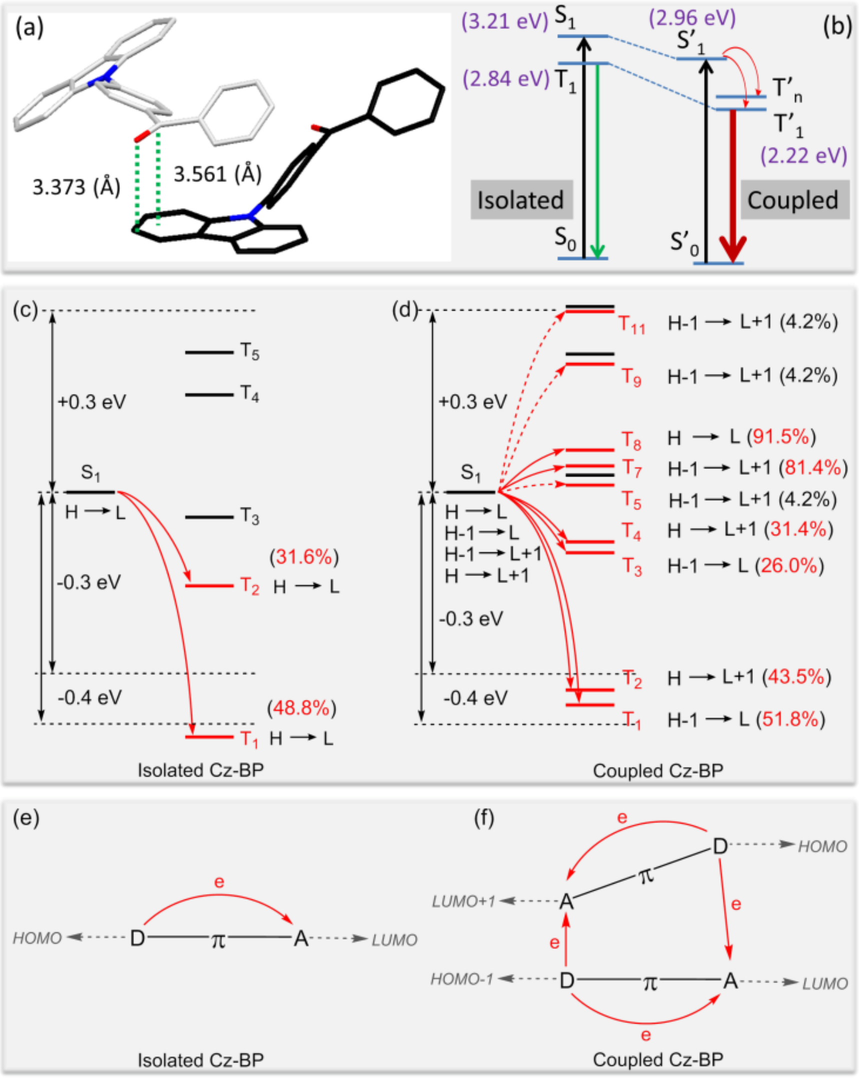

Figure 3. (a) Intermolecular coupling of the carbonyl and Cz groups in two Cz-BP molecules that are in close proximity in a single crystal. (b) Energy level diagram of the isolated and coupled Cz-BP molecule(s). Schematic representations of the TD-DFT calculated energy levels, main orbital configurations and possible ISC channels of (c) isolated Cz-BP and (d) coupled Cz-BP at the singlet (S_1) and triplet (T_n) states. H and L refer to HOMO and LUMO, respectively. Schematic representations of the possible intramolecular and intermolecular ISC channels in (e) isolated Cz-BP and (f) coupled Cz-BP molecules. The carbonyl (electron acceptor) and the Cz (electron donor) units are labeled as A and D, respectively. The plain and dashed arrows refer to the major and minor ISC channels in (c), (d), (e), and (f), respectively.

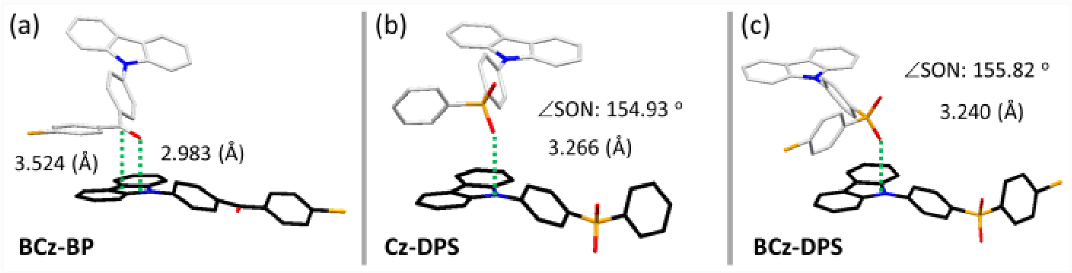

Figure 4. Intermolecular coupling of the n and π units in two neighboring molecules in single crystal structures: (a) Cz-DPS, (b) BCz-BP, and (c) BCz-DPS. The distance from the n group to the coupled π plane Cz plane) is indicated by green dashed lines: (a) 2.983 Å (O−Cz plane) and 3.524 Å (C−Cz plane), (b) 3.266 Å (O−Cz plane), and (c) 3.240 Å (O−Cz plane). The relative angle (∠SON) between the S=O bond and the N atom of the Cz plane in (b) and (c) is also indicated: (b) 154.93 ^{o} and (c) 155.82 ^{o}.

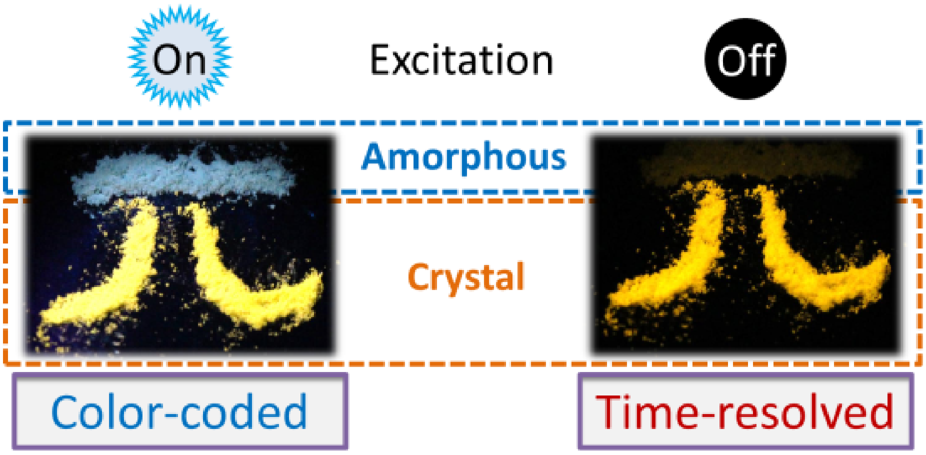

Figure 5. Two-dimensional security protection applications involving the use of BCz-BP for color-coded and time-resolved applications. When excited with 365 nm ultraviolet irradiation, the ground part (light blue) of security letter 'π' was clearly recognized from the crystal part (yellow). After the excitation is turned off, only the crystal part (orange) of the letter 'π' can be observed.

References

1 Z. Mao, Z. Yang, Y. Mu, Y. Zhang, Y.-F. Wang, Z. Chi, C.-C. Lo, S. Liu, A. Lien, J. Xu, *Angew. Chem. Int. Ed.* **2015,** *54*, 6270–6273; *Angew. Chem.* **2015**,

127, 6368−6371.

2 M. Montalti, A. Credi, L. Prodi, M. T. Gandolfi, *Handbook of Photochemistry*, CRC Press, **2006**.

3 Z. An, C. Zheng, Y. Tao, R. Chen, H. Shi, T. Chen, Z. Wang, H. Li, R. Deng, X. Liu, W. Huang, *Nat. Mater.* **2015**, Doi: 10.1038/nmat4259.

4 P. Xue, J. Sun, P. Chen, P. Wang, B. Yao, P. Gong, Z. Zhang, R. Lu, *Chem. Commun.* **2015**, *51*, 10381−10384.

5 C. Li, X. Tang, L. Zhang, C. Li, Z. Liu, Z. Bo, Y. Q. Dong, Y.-H. Tian, Y. Dong, B. Z. Tang, *Adv. Opt. Mater*. **2015**, Doi: 10.1002/adom.201500115.

6 C. S. Bilen, N. Harrison, D. J. Morantz, *Nature* **1978**, *271*, 235−237.

7 Y. Gong, G. Chen, Q. Peng, W. Z. Yuan, Y. Xie, S. Li, Y. Zhang, B. Z. Tang, *Adv. Mater.* **2015**, DOI: 10.1002/adma.201502442.

8 P. de Sainte Claire, *J. Phys. Chem. B* **2006**, *110*, 7334−7343.

9 M. J. Frisch, G. W. Trucks, H. B. Schlegel, G. E. Scuseria, M. A. Robb, J. R.; Scalmani, G. Cheeseman, V. Barone, B. Mennucci, G. A. Petersson, H. Nakatsuji, M. Caricato, X. Li, H. P. Hratchian, A. F. Izmaylov, J. Bloino, G. Zheng, J. L.; Hada, M. Sonnenberg, M. Ehara, K. Toyota, R. Fukuda, J. Hasegawa, M. Ishida, T. Nakajima, Y. Honda, O. Kitao, H. Nakai, T. Vreven, J. A. Jr. Montgomery, J. E. Peralta, F. Ogliaro, M. Bearpark, J. J. Heyd, E. Brothers, K. N. Kudin, V. N. Staroverov, R. Kobayashi, J. Normand, K. Raghavachari, A. Rendell, J. C. Burant, S. S. Iyengar, J. Tomasi, M. Cossi, N. Rega, J. M. Millam, M. Klene, J. E. Knox, J. B. Cross, V. Bakken, C. Adamo, J. Jaramillo, R. Gomperts, R. E. Stratmann, O. Yazyev, A. J. Austin, R. Cammi, C. Pomelli, J. W. Ochterski, R. L. Martin, K. Morokuma, V. G. Zakrzewski, G. A. Voth, P. Salvador, J. J. Dannenberg, S. Dapprich, A. D. Daniels, Ö. Farkas, J. B. Foresman, J. V. Ortiz, J. Cioslowski, D. J. Fox, *Gaussian 09, Revision A.01*, Gaussian, Inc., Wallingford CT, 2009.

10 R. Bauernschmitt, R. Ahlrichs, *Chem. Phys. Lett.* **1996**, *256*, 454−464.

11 a) A. D. Becke, *J. Chem. Phys.* **1993**, *98*, 1372–1377; b) C. Y. Lee, W. Yang, R. G. Parr, *Phys. Rev. B* **1988**, *37*, 785–789.
